# Destruction of DNA‐Binding Proteins by Programmable Oligonucleotide PROTAC (O'PROTAC): Effective Targeting of LEF1 and ERG

**DOI:** 10.1002/advs.202102555

**Published:** 2021-08-16

**Authors:** Jingwei Shao, Yuqian Yan, Donglin Ding, Dejie Wang, Yundong He, Yunqian Pan, Wei Yan, Anupreet Kharbanda, Hong‐yu Li, Haojie Huang

**Affiliations:** ^1^ Department of Pharmaceutical Sciences College of Pharmacy University of Arkansas for Medical Sciences Little Rock AR 72205 USA; ^2^ Department of Biochemistry and Molecular Biology Mayo Clinic College of Medicine and Science Rochester MN 55905 USA; ^3^ Department of Urology Mayo Clinic College of Medicine and Science Rochester MN 55905 USA; ^4^ Mayo Clinic Cancer Center Mayo Clinic College of Medicine and Science Rochester MN 55905 USA

**Keywords:** cancer, oligonucleotide‐based proteolysis‐targeting chimera, protein degradation, transcription factor

## Abstract

DNA‐binding proteins, including transcription factors (TFs), play essential roles in various cellular processes and pathogenesis of diseases, deeming to be potential therapeutic targets. However, these proteins are generally considered undruggable as they lack an enzymatic catalytic site or a ligand‐binding pocket. Proteolysis‐targeting chimera (PROTAC) technology has been developed by engineering a bifunctional molecule chimera to bring a protein of interest (POI) to the proximity of an E3 ubiquitin ligase, thus inducing the ubiquitination of POI and further degradation through the proteasome pathway. Here, the development of oligonucleotide‐based PROTAC (O'PROTACs), a class of noncanonical PROTACs in which a TF‐recognizing double‐stranded oligonucleotide is incorporated as a binding moiety of POI is reported. It is demonstrated that O'PROTACs of lymphoid enhancer‐binding factor 1 (LEF1) and ETS‐related gene (ERG), two highly cancer‐related transcription factors, successfully promote degradation of these proteins, impede their transcriptional activity, and inhibit cancer cell growth in vitro and in vivo. The programmable nature of O'PROTACs indicates that this approach is also applicable to destruct other TFs. O'PROTACs not only can serve as a research tool but also can be harnessed as a therapeutic arsenal to target DNA binding proteins for effective treatment of diseases such as cancer.

## Graphical abstract

DNA binding proteins including transcription factors remain as challenging targets in drug discovery. oligonucleotide‐based proteolysis‐targeting chimeras (O'PROTACs), which employ unique DNA sequences as natural “ligand” of targeting proteins, are developed to effectively destruct lymphoid enhancer‐binding factor 1 (LEF1) and ETS‐related gene (ERG) and inhibit prostate cancer cell growth. O'PROTAC represents an attractive approach for treatment of diseases including cancer.

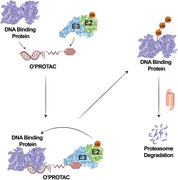

## 1 Introduction

A large group of DNA‐binding proteins acts as transcription factors (TFs) that transcriptionally activate or suppress gene expression by interacting with specific DNA sequence and transcription co‐regulators. ≈2000 TFs have been identified in eukaryotic cells, and they are associated with numerous biological processes. Among them, ≈300 TFs are associated with cancer development, which accounts for ≈19% of oncogenes.^[^ ^1^ ^]^ Therefore, targeting TFs associated with cancer development appears to be a promising strategy for cancer treatment.

In the last decades, small molecule modulators have been developed to target nuclear receptors on the basis that this class of TFs contains a clearly defined ligand‐binding pocket.^[^ ^2^ ^]^ However, most other TFs are challenging to target because they lack a ligand‐binding pocket. As the knowledge regarding the mechanisms of the assembly of transcription complexes has increased exponentially, different strategies to modulate the activity of TFs with small‐molecule compounds have emerged, including blocking protein/protein interactions, protein/DNA interactions, or chromatin remodeling/epigenetic reader proteins.^[^ ^3^ ^]^ However, the development of traditional small molecules inhibiting non‐ligandable TFs still remains very challenging, and a new targeting strategy to overcome the hurdle is highly demanded.

Proteolysis‐targeting chimeras (PROTACs) are heterobifunctional small molecules composed of a protein of interest (POI) ligand as a warhead, a linker, and an E3 ligase ligand. The PROTAC molecule recruits the E3 ligase to the POI and induces the ubiquitination of the latter and further degradation by the proteasome pathway.^[^ ^4^ ^]^ PROTAC technology has significantly advanced during the last decade. It has been reported that PROTACs are capable of degrading more than 100 targets, and a few PROTACs have entered clinical trials.^[^ ^5^ ^]^ PROTACs offer several advantages over small molecule inhibitors, including expanding target scope, improving selectivity, reducing toxicity, and evading inhibitor resistance.^[^ ^6^ ^]^ This suggests that PROTAC technology is a new promising modality to tackle diseases, especially cancer. Most recently, PROTACs have been designed to degrade TFs. In 2019, Wang's group developed potent and signal transducers and activators of transcription 3 (STAT3)‐specific degrader based on the STAT3 inhibitor SI‐109 and demonstrated its targeting efficacy in vivo.^[^ ^7^ ^]^ Recently, Crews’ group reported the development of transcription factor targeting chimeras (TRAFTACs),^[^ ^8^ ^]^ which utilize haloPROTAC, dCas9‐HT7, and dsDNA/CRISPR‐RNA chimeras to degrade TFs. Nevertheless, this approach uses the artificially engineered dCas9‐HT7 fusion protein as a mediator, limiting its potential use in the clinic.

Lymphoid enhancer‐binding factor 1 (LEF1) is a highly cancer‐related TF. It belongs to the T‐cell factor (TCF)/LEF1 family. Complexed with *β*‐catenin, LEF1 promotes the transcription of Wnt target genes.^[^ ^9^ ^]^ LEF1 also can facilitate epithelial‐mesenchymal transition (EMT).^[^ ^10^ ^]^ Aberrant expression of LEF1 is implicated in several cancer types and related to cancer cell proliferation, migration, and invasion.^[^ ^11^ ^]^ Therefore, therapeutic targeting LEF1 is urgently necessary to treat prostate cancer patients effectively.

ETS‐related gene (ERG) transcription factor belongs to the erythroblast transformation‐specific (ETS) family and is involved in bone development, hematopoiesis, angiogenesis, vasculogenesis, inflammation, migration, and invasion.^[^ ^12^ ^]^ It is significantly overexpressed in nearly 50% of all human prostate cancer cases, including primary and metastatic prostate cancer, due to the fusion of the *ERG* gene with the androgen‐responsive *TMPRSS2* gene promoter.^[^ ^13^ ^]^ *TMPRSS2‐ERG* gene fusion results in aberrant overexpression of truncated ERG, implying that increased expression of ERG is a pivotal factor to drive prostate cancer progression.^[^ ^14^ ^]^ Hence, ERG is another attractive target for prostate cancer treatment.

**Figure 1:**
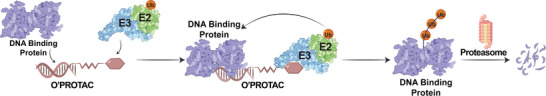
Schematic depicting the working principle of O'PROTAC.

In the present study, we introduce a new strategy to target TFs using O'PROTACs, in which a double‐stranded oligonucleotide is incorporated as POI binding moiety in PROTAC (**Figure** **1**). We demonstrate that LEF1 O'PROTAC promotes proteasomal degradation of LEF1 protein and inhibits LEF1 transcriptional activity and prostate cancer cell growth in vitro and in mice. Akin to LEF1 degrader, ERG O'PROTAC induces the degradation of ERG and inhibits prostate cancer cell growth.

## 2 Results

### 2.1 Design of LEF1 O'PROTACs

**Figure 2:**
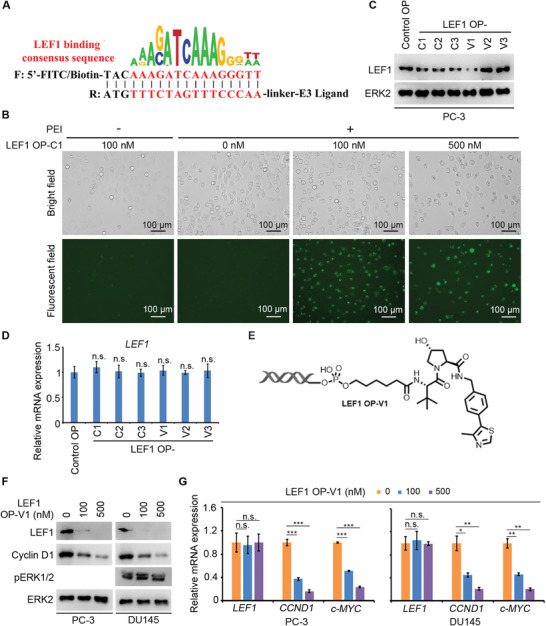
LEF1 O'PROTACs degrade LEF1 protein in cultured cells. A) A schematic diagram for LEF1 O'PROTACs. B) FITC‐labeled LEF1 O'PROTACs, including LEF1 OP‐C1 to C3 and OP‐V1 to V3, were transfected individually with PEI into PC‐3 cells in different doses. Representative images of bright and fluorescent fields for OP‐C1 are shown. C,D) PC‐3 cells were transfected with control or six indicated LEF1 O'PROTACs (100 nm). Cells were collected for western blot analysis (C) or evaluation of *LEF1* mRNA level (D) after 48 h transfection. Data represents means ± SD (*n* = 3). *P* values were determined using the unpaired two‐tailed Student's *t*‐test. n.s., not significant comparing the values in the LEF1 O'PROTAC‐treated groups to that in the group treated with the control OP. E) A schematic diagram for LEF1 OP‐V1. F) The protein level of LEF1, Cyclin D1, and phosphorylated ERK1/2 (pERK1/2) was examined by western blot in PC‐3 (except pERK1/2) and DU145 cells at 48 h after transfection with LEF1 OP‐V1 at different concentrations. ERK2 was used as a loading control. G) The mRNA level of *LEF1*, *CCND1*, and *MYC* genes was analyzed by RT‐qPCR in PC‐3 and DU145 cells at 48 h after transfection with LEF1 OP‐V1. Data represents means ± SD (*n* = 3). *P* values were determined using the unpaired two‐tailed Student's *t*‐test. n.s., not significant; * *P* < 0.05; ** *P* < 0.01; *** *P* < 0.001.

Transcription factors directly control gene expression by recognizing specific DNA sequences, which has been adequately summarized in a previous report.^[^ ^15^ ^]^ LEF1 acts as a DNA binding subunit in the *β*‐catenin/LEF1 complex and exerts transcriptional regulation via binding to the nucleotide sequence 5′‐AAAGATCAAAGGGTT‐3′.^[^ ^16^ ^]^ We designed 18‐mer double‐stranded oligonucleotide (TACAAAGATCAAAGGGTT)‐based LEF1 O'PROTACs by including the LEF1 binding moiety (underscored) and three extra nucleotides for protection of oligo degradation (**Figure** **2**A). A non‐specific sequence (TGTGCTAGCTGATGTGCTA) was chosen as control based on the in silico prediction by the PROMO software (version 8.3 of TRANSFAC) to ensure that no known proteins enable to bind to this sequence. As for the E3 ligase‐recruiting element, we selected the widely used pomalidomide and VH 032, which are capable of hijacking cereblon (CRBN) and von Hippel–Lindau (VHL) E3 ligase, respectively.^[^ ^17^ ^]^ We first evaluated CRBN and VHL protein expression in various prostatic cell lines (Figure S1A, Supporting Information) and found that both E3 ligases were well expressed in the most of these cell lines. PROTAC exerts its function based on the formation of a ternary complex, in which a linker plays an important role. Therefore, we designed and synthesized six pomalidomide‐ and VH 032‐based phosphoramidites with linkers in different lengths and types (Scheme S1, Supporting Information). We then attempted to attach the phosphoramidite to the 5′ end of the reverse strand following a conventional phosphoramidite protocol on DNA synthesizer (Scheme S2A, Supporting Information). After annealing with forward strand, we generated six oligonucleotide‐based PROTACs (O'PROTACs or OPs) for LEF1. The first three O'PROTACs utilized CRBN ligand and linker length of 5, 8, and 11 atoms (termed LEF1 OP‐C1 to C3) and the remaining three O'PROTACs used VHL ligand and linker length of 6, 8, and 11 atoms (termed LEF1 OP‐V1 to V3) (Tables S1 and S2, Supporting Information).

### 2.2 Identification of LEF1 OP‐V1 as a Degrader of LEF1 Protein

Through robust purification by high performance liquid chromatography (HPLC) and mass spectrometry analysis, we found that VH 032‐based O'PROTACs (V1 to V3) were successfully constructed with high purity (>95%); however, the purity of pomalidomide‐based O'PROTACs (C1 to C3) was much lower. The nucleic acid‐related agents can be delivered into cells via lipid‐based approaches or other means. Fluorescein isothiocyanate (FITC) was incorporated in LEF1 O'PROTACs to determine the transfection efficiency. These FITC‐labeled LEF1 O'PROTACs were transfected into PC‐3 prostate cancer cells, which express endogenous LEF1 (Figure S1A, Supporting Information). In agreement with the purity analysis, we found that none of FITC‐labeled pomalidomide‐based LEF1 O'PROTACs (LEF1 OP‐C1 to C3) was potent to degrade LEF1 in PC‐3 cells, although these O'PROTACs were effectively transfected into cells (Figure 2B,C). In contrast, LEF1 OP‐V1, but not OP‐V2 and OP‐V3 (all FITC‐labeled) enabled to effectively downregulate LEF1 at protein level (Figure 2C), suggesting that the VHL ligand‐derived LEF1 O'PROTAC with a shorter linker favors the stable ternary complex and promotes protein degradation. This notion is further supported by the observation that LEF1 OP‐V1 had no effect on LEF1 expression at mRNA level (Figure 2D). These data indicate that such effect occurs at post‐transcription level. Therefore, we chose LEF1 OP‐V1 (Figure 2E) for further biochemical and functional studies.

We further verified the effectiveness of LEF1 OP‐V1 in DU145, another LEF1‐expressing prostate cancer cell line (Figure S1A, Supporting Information). Similar to the effect in PC‐3 cells, OP‐V1 also induced the degradation of LEF1 in DU145 (Figure 2F). We also investigated the effect of LEF1 OP‐V1 on the transcriptional activity of the *β*‐catenin/LEF1 complex. We found that LEF1 OP‐V1 treatment downregulated mRNA expression of *CCND1* and *c‐MYC*, two known target genes of *β*‐catenin/LEF1 in both PC‐3 and DU145 cells (Figure 2G). Thus, LEF1 OP‐V1 can not only downregulate LEF1 protein, but also inhibit its transcriptional activity in prostate cancer cells.

**Figure 3:**
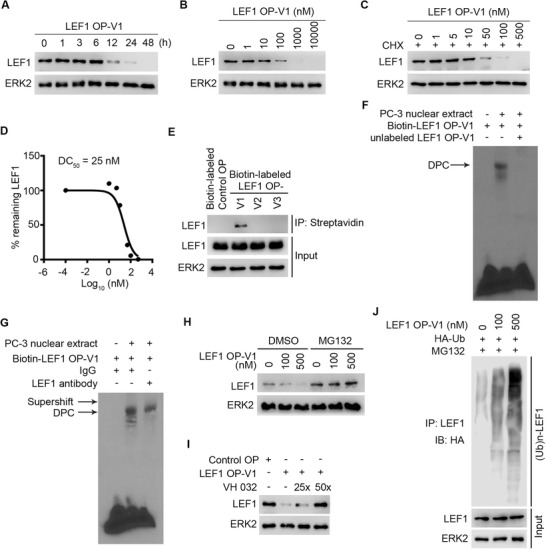
LEF1 OP‐V1 O'PROTAC effectively binds to and degrades LEF1 protein through ubiquitin–proteasome system. A) PC‐3 cells were transfected with 100 nm LEF1 OP‐V1 and collected at indicated time points for western blot analysis of LEF1 protein expression. B) PC‐3 cells were transfected with different doses of LEF1 OP‐V1 for 36 h, followed by western blot analysis of LEF1 protein expression. C,D) PC‐3 cells were transfected with increasing concentrations of LEF1 OP‐V1 for 24 h and treated with 20 µg mL^−1^ cycloheximide (CHX) for another 12 h. Cells were harvested for western blot analysis of LEF1 protein expression (C). The remaining LEF1 protein (%) was calculated by normalizing the value in each group to that in the group without LEF1 OP‐V1 treatment, and DC_50_ was determined (D). This experiment was repeated once and similar results were obtained. E) PC‐3 cells were transfected with biotin‐labeled control or three indicated LEF1 O'PROTACs (100 nm) and treated with MG132 (20 µg mL^−1^). Cells were harvested for anti‐biotin (streptavidin) pulldown assay 24 h post transfection. F) Biotin‐labeled LEF1 OP‐V1 was incubated with PC‐3 nuclear extract in the presence or absence of unlabeled counterpart (100‐fold of biotin‐labeled probe) followed by EMSA. DPC stands for DNA‐protein complex. G) Biotin‐labeled LEF1 OP‐V1 was incubated with PC‐3 nuclear extract in the presence or absence of LEF1 antibody followed by EMSA. H) PC‐3 cells were subjected to western blot analysis after transfected with LEF1 OP‐V1 (100 or 500 nm) for 48 h in the presence or absence of MG132 (20 µm). I) PC‐3 cells were transfected with either control OP or LEF1 OP‐V1 (100 nm) in the presence of different fold (25×, 50×) of VHL ligand VH 032. J) PC‐3 cells were co‐transfected with LEF1 OP‐V1 (100 or 500 nm) and HA‐Ub. Cells were collected after 48 h for LEF1 ubiquitination analysis.

To evaluate the kinetics of LEF1 degradation, we performed the time course experiment in PC‐3 cells (**Figure** **3**A). Substantial degradation of LEF1 protein was observed starting at 12 h and the effect remained through 48 h. We also determined the dose effect of OP‐V1 on LEF1 protein destruction in PC‐3 cells (Figure 3B). LEF1 OP‐V1 induced LEF1 protein degradation in a dose‐dependent manner, with the DC_50_ (50% degradation) value of 25 nm (Figure 3C,D). Taken together, LEF1 OP‐V1 degrades LEF1 protein in a time‐ and dose‐dependent manner.

### 2.3 LEF1 O'PROTAC Effectively Binds To and Degrades LEF1 Protein through Ubiquitin–Proteasome System

To explore the mechanism of action of LEF1 OP‐V1 on LEF1 protein expression, we first performed biotin pulldown assay using biotin‐labeled OP‐V1 to V3. Consistent with the effect of LEF1 OP‐V1 on LEF1 protein downregulation, we demonstrated that biotin‐labeled LEF1 OP‐V1 strongly bound to endogenous LEF1 in PC‐3 cells compared to other two LEF‐1 O'PROTACs examined (Figure 3E). This observation was further substantiated by endogenous LEF1 immunofluorescence data (Figure S1B, Supporting Information). The electrophoretic mobility shift assay (EMSA) confirmed that LEF1 OP‐V1 could form a DNA‐protein complex (DPC) in the nuclear extract of PC‐3 cells. This binding appears to be LEF1 specific since addition of non‐biotin‐labeled LEF1 OP‐V1 completely blocked DPC formation and incubation of the reaction with anti‐LEF1 antibody resulted in a supershift of the DPC (Figure 3F,G). We provided evidence that the effective LEF1 O'PROTAC can bind to LEF1.

Next, we determine whether O'PROTAC‐induced downregulation of LEF1 proteins was mediated through proteasome degradation. We demonstrated that LEF1 OP‐V1‐induced degradation of LEF1 proteins was completely blocked by the proteasome inhibitor MG132 (Figure 3H). These data suggest that the downregulation of LEF1 induced by LEF1 OP‐V1 is mediated through the proteasome pathway. Furthermore, pretreatment with excessive VHL ligand VH 032 abolished the degradation of LEF1 by OP‐V1 (Figure 3I), suggesting that the effect of LEF1 OP‐V1 on LEF1 degradation is mediated through VHL. Moreover, we showed that LEF1 OP‐V1 treatment effectively increased LEF1 protein poly‐ubiquitination in PC‐3 cells (Figure 3J). Thus, LEF1 OP‐V1 induces the ubiquitination and proteasomal degradation of LEF1 protein in VHL‐dependent manner.

### 2.4 LEF1 OP‐V1 Inhibits Prostate Cancer Cell Proliferation In Vitro and Tumor Growth In Vivo

**Figure 4:**
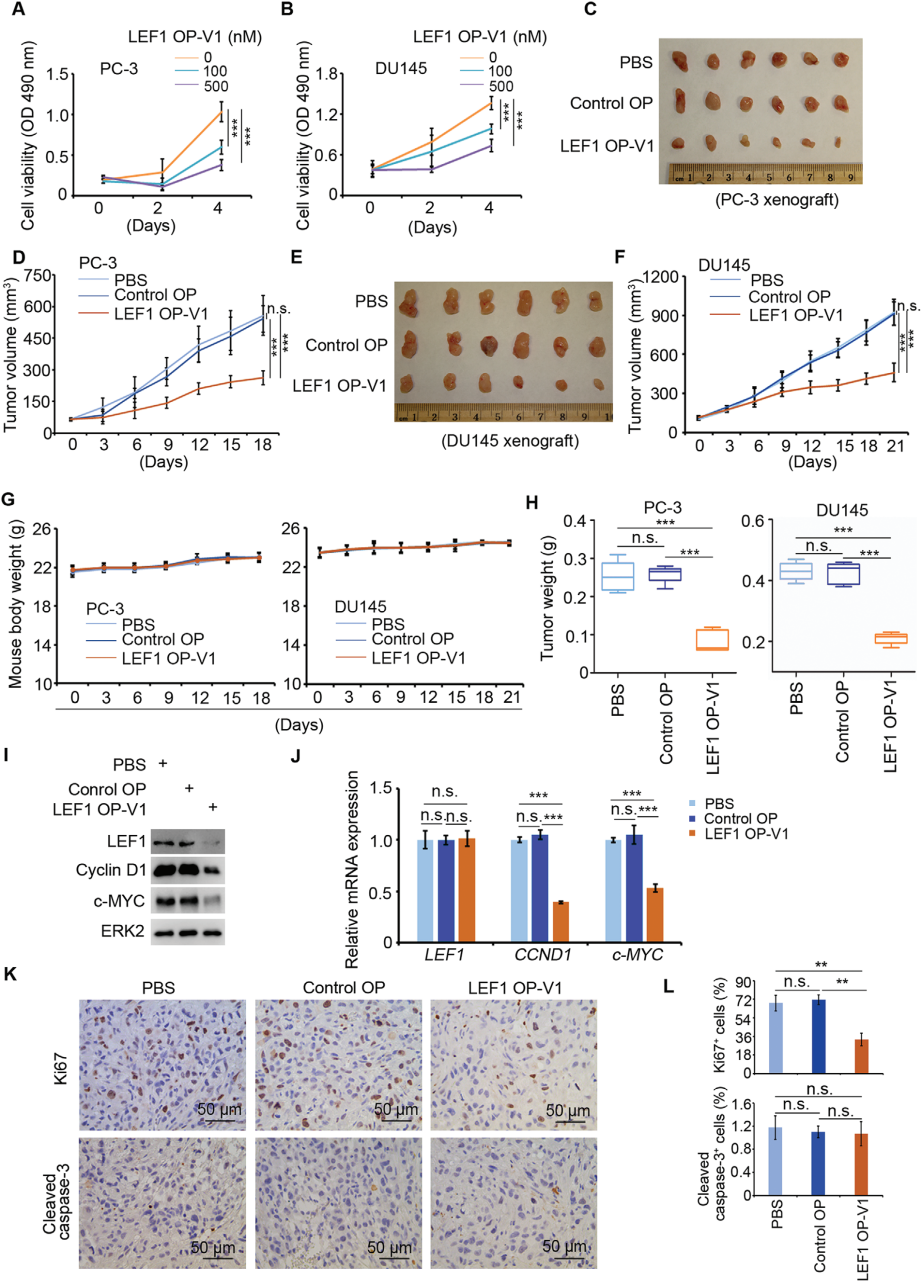
LEF1 OP‐V1 inhibits prostate cancer cell invasion and proliferation in vitro and tumor growth in vivo. A,B) PC‐3 and DU145 cells transfected with LEF1 OP‐V1 at the indicated concentrations were subjected to MTS assay at the indicated time points. Data represents means ± SD (*n* = 5). *P* values were determined using the unpaired two‐tailed Student's *t*‐test at day 4. *** *P* < 0.001. C) Photos of PC‐3 xenograft tumors from the indicated groups of mice at 18 days after treatment with 1 × PBS, control OP or LEF1 OP‐V1. D) PC‐3 tumor growth was measured at indicated time points after treatment with 1 × PBS, control OP or LEF1 OP‐V1. Data represents means ± SD (*n* = 6). *P* values were determined using the unpaired two‐tailed Student's *t*‐test at day 18. n.s., not significant; *** *P* < 0.001. E) Photos of DU145 xenograft tumors from the indicated groups of mice at 21 days after treatment with 1 × PBS, control OP or LEF1 OP‐V1. F) DU145 tumor growth was measured at indicated time points after treatment with 1 × PBS, control OP or LEF1 OP‐V1. Data represents means ± SD (*n* = 6). *P* values were determined using the unpaired two‐tailed Student's *t*‐test at day 18. n.s., not significant; *** *P* < 0.001. G) Body weight of mice was measured at different time points after the indicated treatments in PC‐3 or DU145 xenograft. Data represents means ± SD (*n* = 6). (H) PC‐3 and DU145 xenograft tumors were harvested from mice at days 18 or 21, respectively and their weight was measured. Data represents means ± SD (*n* = 6). *P* values were determined using the unpaired two‐tailed Student's *t*‐test. n.s., not significant; *** *P* < 0.001. I) Western blot analysis of expression of LEF1, cyclin D1, and c‐MYC protein in PC‐3 xenograft tumors. J) RT‐qPCR analysis of mRNA level of *LEF1*, *CCND1*, and *c‐MYC* genes in PC‐3 xenograft tumors. Data represents means ± SD (*n* = 6). *P* values were determined using the unpaired two‐tailed Student's *t*‐test. n.s., not significant; *** *P* < 0.001. K) Representative images of IHC of LEF1, Ki67, and cleaved caspase‐3 in PC‐3 xenograft tumors harvested from mice at 18 days after treatment with 1 × PBS, control OP, or LEF1 OP‐V1. L) The quantification data of the LEF1, Ki67, and cleaved caspase‐3 IHC. Data represents means ± SD (*n* = 6). *P* values were determined using the unpaired two‐tailed Student's *t*‐test. n.s., not significant; *** *P* < 0.001.

Having identified OP‐V1 as a potent LEF1 degrader, we explored the anti‐cancer efficacy of this O'PROTAC. The growth of both PC‐3 and DU145 cells was significantly inhibited by LEF1 OP‐V1 in vitro (**Figure** **4**A,B). We further investigated the effect of LEF OP‐V1 in vivo. PC‐3 and DU145 xenograft tumors were generated by subcutaneous injection of PC‐3 and DU145 cells into SCID mice. By treating mice with positively charged polyethylenimine (PEI)‐condensed DNA oligo‐based O'PROTAC, we demonstrated that LEF1 OP‐V1 effectively inhibited PC‐3 and DU145 tumor growth in mice compared to the treatment of phosphate‐buffered saline (PBS) or control OP (Figure 4C–F). Little or no pronounced effect was observed on the weight loss of mice after administration of LEF1 OP‐V1 (Figure 4G). On the contrary, the tumor weight was largely decreased by the treatment of LEF1 OP‐V1 (Figure 4H), implying the inhibitory effect of LEF1 OP‐V1 on tumor growth was not caused by the general toxicity of the O'PROTAC in mice. Consistent with the effect of LEF1 OP‐V1 on tumor growth, LEF1 OP‐V1 treatment decreased LEF1 protein and inhibited LEF1/*β*‐catenin target gene expression in tumors (Figure 4I,J). Importantly, LEF1 OP‐V1 treatment also significantly impeded Ki67 expression in PC‐3 tumors we examined, although little or no noticeable effect of LEF1 OP‐V1 on cell death was observed (Figure 4K,L). These data suggest that we have successfully identified a LEF1 O'PROTAC that can effectively deplete LEF1 protein and inhibit prostate cancer cell growth in vivo.

### 2.5 ERG O'PROTAC Induces ERG Protein Degradation

**Figure 5:**
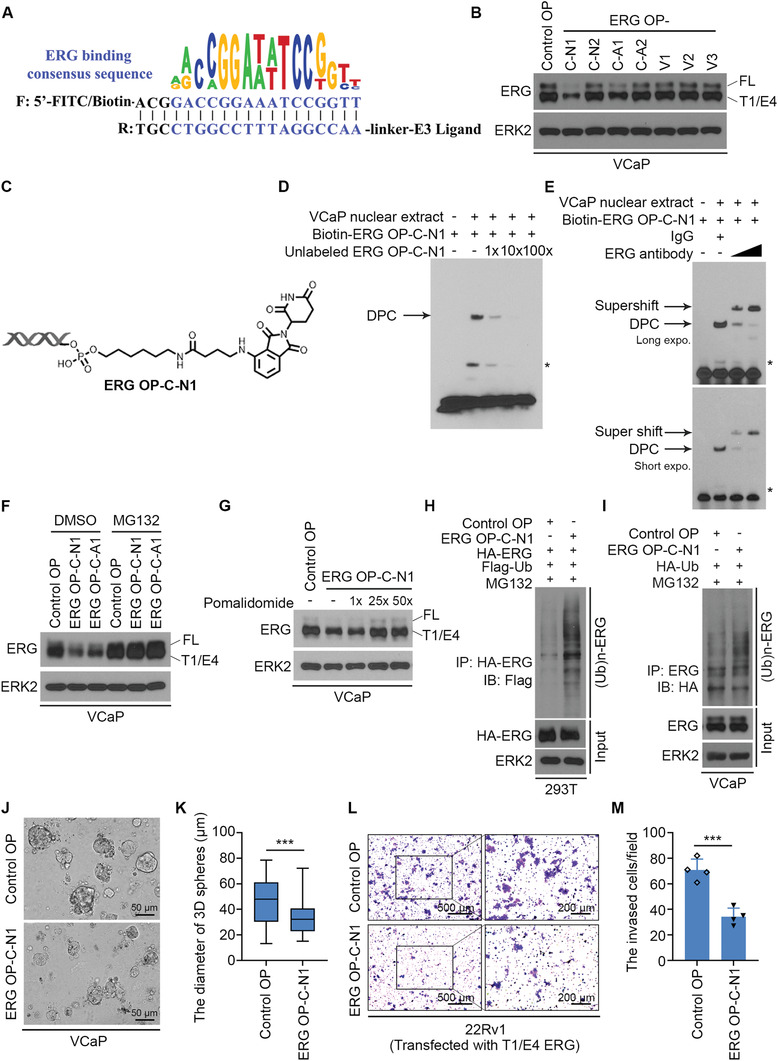
ERG O'PROTAC induces ERG protein degradation. A) A schematic diagram for ERG O'PROTACs. B) VCaP cells were transfected with control or seven indicated ERG O'PROTACs (100 nm) for 36 h and harvested for western blot analysis. ERK2 was used as a loading control. C) A schematic diagram for ERG OP‐C‐N1 structure. D) Biotin‐labeled ERG OP‐C‐N1 (100 nm) was incubated with VCaP nuclear extract in the presence of an increasing amount of the unlabeled counterparts (1‐, 10‐, and 100‐fold higher than the concentration of biotin‐labeled probe) followed by EMSA. E) Biotin‐labeled ERG OP‐C‐N1 was incubated with VCaP nuclear extract and an increasing amount of ERG antibody, followed by EMSA. F) VCaP cells were transfected with control OP, ERG OP‐C‐N1 (100 nm) or OP‐C‐A1 (100 nm) for 36 h, followed by treatment of the proteasome inhibitor MG132 (20 µm) for 12 h and western blot analysis. G) VCaP cells were transfected with control OP or ERG OP‐C‐N1 at a final concentration of 100 nm for 36 h and incubated with 1‐, 25‐, or 50‐fold of CRBN ligand pomalidomide, followed by western blot analysis of ERG expression. H,I) 293T (H) and VCaP cells (I) were treated with ERG OP‐C‐N1 (100 nm) for 36 h and the proteasome inhibitor MG132 (20 µm) for 12 h before harvested for western blot analysis of ERG ubiquitination. J,K) VCaP cells were cultured in Matrigel for 5 days followed by the treatment of 200 nm of ERG OP‐C‐N1 for another 5 days. The representative images with 3D spheres are shown in (J) and the quantification data of the diameters of the 3D spheres are shown in (K). Data represents means ± SD (*n* = 50). *P* values were determined using the unpaired two‐tailed Student's *t*‐test; *** *P* < 0.001. L,M). 22Rv1 cells transfected with ERG expression plasmid and 100 nm of ERG OP‐C‐N1 were plated onto Matrigel‐coated transwells for 48 h. The invaded cells were stained with crystal violet. Photos are shown in (L) and the quantification data are shown in (M). Data represents means ± SD (*n* = 4). *P* values were determined using the unpaired two‐tailed Student's *t*‐test. *** *P* < 0.001.

To assess the feasibility of the O'PROTAC strategy in degrading other TFs, we designed and synthesized O'PROTACs to destruct ERG, which is overexpressed in ≈50% of prostate cancer cases in patients. ERG recognizes a highly specific DNA sequence 5′‐GACCGGAAATCCGGTT‐3′ core motif.^[^ ^18^ ^]^ We designed a 19‐mer double‐stranded oligonucleotide (ACGGACCGGAAATCCGGTT)‐based ERG O'PROTACs by including ERG binding moiety (underscored) and three extra nucleotides for protection of oligo degradation (**Figure** **5**A and Table S2, Supporting Information). Similar to the scenario of LEF1 O'PROTACs synthesized by phosphoramidite chemistry, the purity of ERG OP‐V1 to V3 was very high (>95%), but OP‐C1 to C3 was not optimal. Because none of the VHL ligand‐based ERG O'PROTACs (OP‐V1 to V3) was effective in degrading ERG in VCaP cells which express both endogenous WT and TMPRSS2‐ERG truncation (TMPRSS2 exon 1 (T1) fused with ERG exon 4 (E4) or termed T1/E4) proteins (Figure 5B), we employed post‐synthesis conjugations to develop more pomalidomide‐based O'PROTAC. NHS‐ester and azide intermediates were synthesized and subsequently incorporated to oligonucleotide through NHS‐ester modification and click reaction, respectively (Scheme S2B,C, Supporting Information). Ultimately, we generated four ERG pomalidomide ‐based PROTACs (termed OP‐C‐N1, OP‐C‐N2, OP‐C‐A1, and OP‐C‐A2) (Table S2, Supporting Information).

Among these ERG O'PROTACs generated through post‐synthesis conjugations, two of them (OP‐C‐N1 and OP‐C‐A1), especially ERG OP‐C‐N1 potently degraded ERG protein in VCaP cells (Figure 5B). As with LEF1 OP‐V1, we chose ERG OP‐C‐N1 (Figure 5C) for further investigation. The kinetics experiment confirmed that ERG OP‐C‐N1 effectively degraded ERG protein in a time‐ and dose‐dependent manner (Figure S2A,B, Supporting Information). Moreover, we showed that the DC_50_ of ERG OP‐C‐N1 is 182.4 nm (Figure S2C,D, Supporting Information). We examined whether ERG OP‐C‐N1 can bind to ERG in vitro, by performing EMSA using nuclear extract of VCaP cells. We demonstrated that biotin‐labeled ERG OP‐C‐N1 formed a DPC after incubation with VCaP nuclear extract. This binding was abolished by the addition of competitive non‐biotin‐labeled ERG OP‐C‐N1 (Figure 5D). Addition of ERG antibody resulted in a supershift of DPC (Figure 5E), suggesting that the detected DPC contains ERG protein. Furthermore, we showed that the destabilization of ERG protein by OP‐C‐N1 was abolished by pretreatment with MG132 (Figure 5F) and pomalidomide (Figure 5G). In agreement with this observation, we demonstrated that ERG OP‐C‐N1 treatment increased poly‐ubiquitination of ERG protein (Figure 5H,I). To identify the anti‐cellular effect of ERG OP‐C‐N1, we performed 3D culture for VCaP cells after the treatment of ERG OP‐C‐N1. The quantification of 3D culture diameter showed that ERG OP‐C‐N1 inhibited VCaP cell growth in vitro (Figure 5J,K). Moreover, cell invasion assay showed that the treatment of OP‐C‐N1 decreased the invasion ability of VCaP cells (Figure 5L,M). Thus, we identify a bioactive ERG O'PROTAC that can degrade ERG protein and inhibit cancer cell growth in vitro.

## 3 Discussion

Building upon our reporting of the previously uncharacterized strategy of O'PROTACs to destruct generally “undruggable” transcription factors in bioRxiv,^[^ ^19^ ^]^ in the current communication we systematically present the procedures of design and synthesis of LEF1 and ERG O'PROTACs, the mechanisms of action, and their anti‐cancer efficacy in vitro and in vivo. O'PROTAC is designed based on natural “ligand” of transcription factors, namely specific DNA sequence, attached to an E3 ligase ligand via a linker. Each TF recognizes fairly unique DNA sequences. Although transcription factors typically bind to the core sequence, usually ≈10 nt in length,^[^ ^20^ ^]^ the flanking bases confers the additional substrate specificity.^[^ ^21^ ^]^ Therefore, from those LEF1 and ERG respective binding sequences, the longest sequences^[^ ^16^, ^18^ ^]^ were chosen as their binding motifs and expected to have better selectivity. Besides, three additional bases are added to 3′ end of the reverse strand to prevent the exonucleases‐mediated hydrolysis.^[^ ^22^ ^]^ Hence, double‐stranded oligonucleotides with length of 18 and 19 bp was used to develop LEF1 and ERG O'PROTACs, respectively. Our data also stress that potency of O'PROTACs varies and likely depends on the lengths and types of a linker as well as the E3 ligase ligand. While the exact underlying mechanisms are unclear at present, a comprehensive picture could emerge with more O'PROTACs designed and explored.

Previous studies indicate that PROTACs can initiate protein degradation very fast, although the time points vary broadly from 1 to 16 h after degrader exposure.^[^ ^23^ ^]^ Our data demonstrated that LEF1 OP‐V1 and ERG OP‐C‐N1 induced the initiation of degradation of LEF1 and ERG proteins at 12 and 24 h, respectively in prostate cancer cell lines examined. Due to the oligo‐based nature of O'PROTACs and because their delivery into cells likely takes longer time than small molecule PROTACs, our data suggest that O'PROTACs may act similarly on protein destruction as the conventional counterparts. Furthermore, hook effect has been observed with many PROTACs.^[^ ^24^ ^]^ The dose‐course experiments indicated that no obvious hook effect was detected for LEF1 OP‐V1. However, ERG OP‐C‐N1 might have certain hook effect, at least at the highest concentration we used (10 000 nm). Given that delivery of O'PROTACs relies on liposomes and polymers, they are limited to be used at very high concentrations such as in the order of micro molars. Therefore, it is unlikely that O'PROTACs could have any massive hook effect, at least not at the concentrations we can deliver into cells.

Conventional PROTAC technology is rapidly evolving, and some of PROTACs are in clinical trials; however, it inherits certain limitations. First, most of the reported PROTACs rely on the existing small molecules as POI targeting warhead, making it difficult to be applied to “undruggable” targets like TFs. Additionally, due to their high molecular weight (up to 1400 Da), PROTACs suffer from poor cell permeability, stability, and solubility.^[^ ^25^ ^]^ In comparison with classic small molecule drugs, PROTACs are significantly less druggable. Whereas O'PROTACs hold enormous potentials to transcend the limitations of conventional PROTACs; because of their modalities, degraders can be rationally programmed according to the DNA binding sequence of a given TF, thus theoretically making it possible to target any TF of interest. Moreover, the synthesis of O'PROTAC is straightforward and efficient, which facilitates the rapid development of an O'PROTAC library for high‐throughput screening of the most potent TF degraders.

Hall and colleagues recently report RNA‐PROTACs,^[^ ^26^ ^]^ which utilize single‐stranded RNA (ssRNA) to recruit RNA‐binding protein (RBP). The binding of RBP with RNA heavily relies on both sequence motif and secondary structure.^[^ ^27^ ^]^ Predicting the interaction between RNA and RBP is challenging due to the high flexibility of RNA.^[^ ^28^ ^]^ However, double‐stranded DNA bears a well‐defined 3D duplex structure; therefore, the protein binding region is accessible and more predictable. Hence, O'PROTAC is programmable by changing the nucleotide sequence that binds protein. Additionally, compared with double‐stranded oligonucleotide, ssRNA is susceptible to deleterious chemical or enzymatic attacks.^[^ ^28b^ ^]^ On the contrary, O'PROTAC is desirable for drug development due to its ready predictability and superior stability.

Oligonucleotide‐based drug development has become mainstream for new drug hunting in the last decade, and several oligonucleotides have been FDA‐approved.^[^ ^29^ ^]^ The catalytic advantage of PROTACs incorporated into oligonucleotide drugs could further advance the field. Moreover, the delivery of oligonucleotide drugs has been improved significantly in recent years, such as nucleic acid chemical modifications, conjugation to cell/tissue‐targeting ligands, and nanoparticle carrier systems.^[^ ^30^ ^]^ Therefore, O'PROTAC can be a complementary drug discovery and development platform to conventional PROTACs to derive clinical candidates and accelerate drug discovery.

In summary, we present a proof‐of‐concept of O'PROTAC by identifying the potent and highly efficacious LEF1 and ERG O'PROTACs in vitro and in vivo. Our findings, especially the in vivo efficacy data lays a solid foundation for the further development of O'PROTAC as a new class of therapy for the treatment of human cancers or other diseases in which transcription factors or other DNA binding proteins play a crucial role.

## Conflict of Interest

The authors declare no conflict of interest.

## Author Contributions

J.S., Y.Y., and D.D. contributed equally to this work. H.H. and H.‐Y.L. contributed to the conception and design; J.S., W.Y., and A.K. contributed to the O'PROTAC synthesis; Y.Y., D.D., D.W., Y.H., and Y.P. contributed to the acquisition of data; H.H., H.‐Y.L., J.S., Y.Y., D.D., W.Y., and A.K. contributed to the preparation of the manuscript.

## Supporting information

Supporting Information

## Data Availability

Data available on request from the authors
