# Supplementary material for: Destruction of DNA‐Binding Proteins by Programmable Oligonucleotide PROTAC (O'PROTAC): Effective Targeting of LEF1 and ERG
Source: Adv Sci (Weinh). 2021 Aug 16;8(20):2102555. doi: 10.1002/advs.202102555 (PMC8529430; doi:10.1002/advs.202102555)
Supplement: Supplementary file 1 — Supporting Information [file ADVS-8-2102555-s001.pdf]

© 2021 Wiley-VCH GmbH

ADVANCED SCIENCE
Open Access

Supporting Information

for *Adv. Sci.,* DOI: 10.1002/advs.202102555

Destruction of DNA-binding proteins by programmable oligonucleotide PROTAC (O'PROTAC): Effective targeting of LEF1 and ERG

Jingwei Shao, Yuqian Yan, Donglin Ding, Dejie Wang, Yundong He, Yunqian Pan, Wei Yan, Anupreet Kharbanda, Hong-yu Li and Haojie Huang**

Supporting Information for

Destruction of DNA-binding proteins by programmable oligonucleotide PROTAC (O’PROTAC): Effective targeting of LEF1 and ERG

Jingwei Shao, Yuqian Yan, Donglin Ding, Dejie Wang, Yundong He, Yunqian Pan, Wei Yan, Anupreet Kharbanda, Hong-yu Li and Haojie Huang**

Dr. J. Shao, Dr. W. Yan, A. Kharbanda, Prof. H.-Y. Li,

Department of Pharmaceutical Sciences, College of Pharmacy, University of Arkansas for Medical Sciences, Little Rock, AR 72205, USA

E-mail: HLi2@uams.edu

Dr. Y. Yan, Dr. D. Dong, Dr. D. Wang, Dr. Y. He, Dr. Y. Pan, Prof. H. Huang

Department of Biochemistry and Molecular Biology, Mayo Clinic College of Medicine and Science, Rochester, MN 55905, USA

E-mail: huang.haojie@mayo.edu

Prof. H. Huang

Department of Urology, Mayo Clinic College of Medicine and Science, Rochester, MN 55905, USA

Mayo Clinic Cancer Center, Mayo Clinic College of Medicine and Science, Rochester, MN 55905, USA.

Table S1. Sequences of control and ERG- and LEF1-bound DNA oligos in O’PROTACs

Name[a)]	DNA sequence	Mass calc.	Mass obs.
LEF1-F	5’-TACAAAGATCAAAGGGTT-3’	5555.7	5555.8
LEF1-F-Biotin	5’-Biotin-TACAAAGATCAAAGGGTT-3’	5991.2	5991.8
LEF1-F-FITC	5’-FITC-TACAAAGATCAAAGGGTT-3’	6122.2	6123.7
LEF1-R	3’-ATGTTTCTAGTTTCCCAA-5’	5439.6	NS[b)]
LEF1-R-V1	3’-ATGTTTCTAGTTTCCCAA-L4-VHL-5’	6045.8	6046.4
LEF1-R-V2	3’-ATGTTTCTAGTTTCCCAA-L5-VHL-5’	6077.8	6078.9
LEF1-R-V3	3’-ATGTTTCTAGTTTCCCAA-L6-VHL-5’	6121.8	6122.9
ERG-F	5’-ACGGACCGGAAATCCGGTT-3’	5837.8	5838.0
ERG-F-Biotin	5’-Biotin-ACGGACCGGAAATCCGGTT-3’	6273.3	6274.0
ERG-R	3’-TGCCTGGCCTTTAGGCCAA-5’	5779.8	NS[b)]
ERG-R-C-N1	3’-TGCCTGGCCTTTAGGCCAA-5’-L7-CRBN-5’	6300.0	6300.6
ERG-R-C-N2	3’-TGCCTGGCCTTTAGGCCAA-5’-L8-CRBN-5’	6342.0	6342.6
ERG-R-C-A1	3’-TGCCTGGCCTTTAGGCCAA-5’-L9-CRBN-5’	6296.0	6296.5
ERG-R-C-A2	3’-TGCCTGGCCTTTAGGCCAA-5’-L10-CRBN-5’	6324.0	6324.3
ERG-R-V1	3’-TGCCTGGCCTTTAGGCCAA-5’-L4-VHL-5’	6386.0	6386.2
ERG-R-V2	3’-TGCCTGGCCTTTAGGCCAA-5’-L5-VHL-5’	6418.0	6418.4
ERG-R-V3	3’-TGCCTGGCCTTTAGGCCAA-5’-L6-VHL-5’	6462.0	6462.5
CTRL-F	5’-TGTGCTAGCTGATGTGCTA-3’	5849.9	5850.3

CTRL-R	3’-ACACGATCGACTACACGAT-5’	5765.8	NS[b)]
CTRL-R-C-N1	3’-ACACGATCGACTACACGAT-L7-CRBN-5’	6286.0	6286.6
CTRL-R-V1	3’-ACACGATCGACTACACGAT-L4-VHL-5’	6372.0	6373.1

[a)] F, forward; R, reverse.
[b)] NS: Not synthesized.

Table S2. Design and composition of O'PROTACs

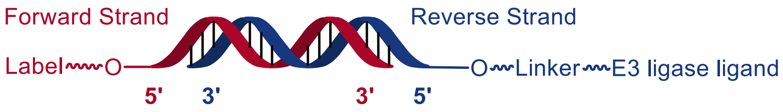

O'PROTAC	Forward (5' to 3')		Reverse (3' to 5') + Linker +E3 ligase ligand			
	Label	Sequence	Sequence	Linker		E3 ligase ligand
				Name	Structure	
**LEF1 OP-C1**	N/A	-LEF1-F-	-LEF1-R-	**L1**	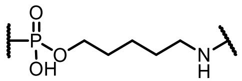	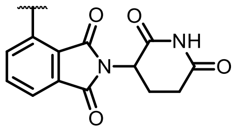 **CRBN**
**LEF1 OP-C2**	N/A	-LEF1-F-	-LEF1-R-	**L2**	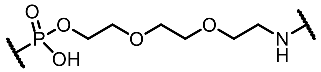	
**LEF1 OP-C3**	N/A	-LEF1-F-	-LEF1-R-	**L3**	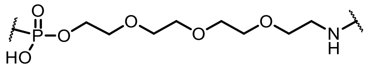	
**LEF1 OP-V1**	N/A	-LEF1-F-	-LEF1-R-	**L4**	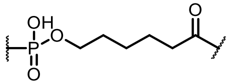	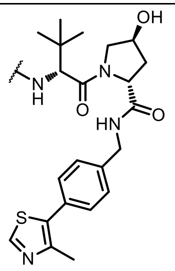 **VHL**
**LEF1 OP-V2**	N/A	-LEF1-F-	-LEF1-R-	**L5**	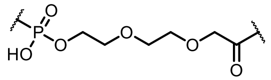	
**LEF1 OP-V3**	N/A	-LEF1-F-	-LEF1-R-	**L6**	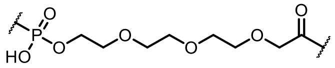	
**LEF1 Biotin-OPs**	-Biotin-	-LEF1-F-	-LEF1-R-	**L1-3 or L4-6**		**CRBN or VHL**
**LEF1 FITC-OPs**	-FITC-	-LEF1-F-	-LEF1-R-	**L1-3 or L4-6**		**CRBN or VHL**

**ERG OP-C1**	N/A	-ERG-F-	-ERG-R-	**L1**	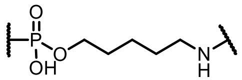	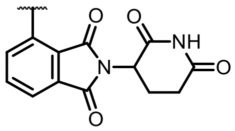
**ERG OP-C2**	N/A	-ERG-F-	-ERG-R-	**L2**	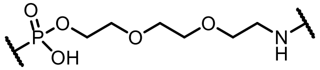	
**ERG OP-C3**	N/A	-ERG-F-	-ERG-R-	**L3**	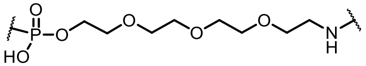	
**ERG OP-C-N1**	N/A	-ERG-F-	-ERG-R-	**L7**	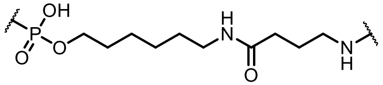	
**ERG OP-C-N2**	N/A	-ERG-F-	-ERG-R-	**L8**	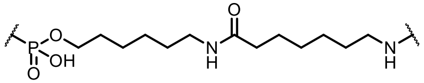	
**ERG OP-C-A1**	N/A	-ERG-F-	-ERG-R-	**L9**	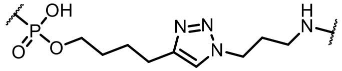	
**ERG OP-C-A2**	N/A	-ERG-F-	-ERG-R-	**L10**	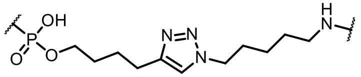	
**ERG OP-V1**	N/A	-ERG-F-	-ERG-R-	**L4**	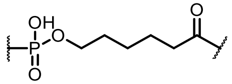	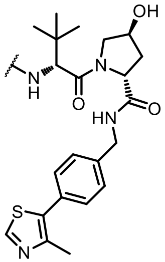
**ERG OP-V2**	N/A	-ERG-F-	-ERG-R-	**L5**	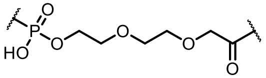	
**ERG OP-V3**	N/A	-ERG-F-	-ERG-R-	**L6**	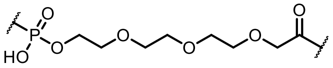	
**ERG Biotin-OPs**	-Biotin-	-ERG-F-	-ERG-R-	**L7-10 or L4-6**		**CRBN or VHL**
**ERG Control OP**	N/A	-CTRL-F-	-CTRL-R-	**L7**	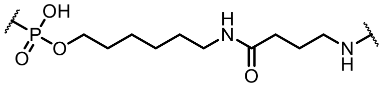	**CRBN**
**LEF1 Control OP**	N/A	-CTRL-F-	-CTRL-R-	**L4**	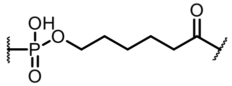	**VHL**

Scheme S1. Synthesis of modifiers[a]

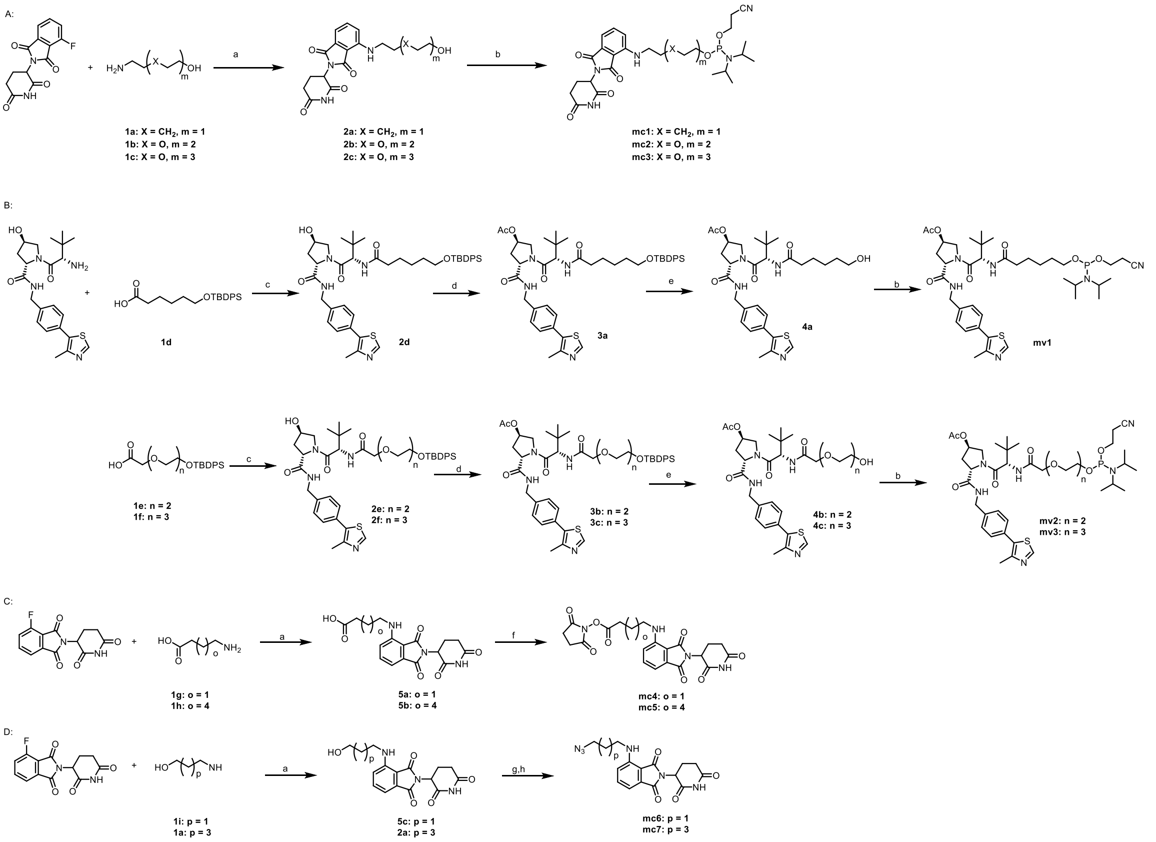

[a]Reagents and conditions: (a) DIPEA, NMP, MW, 100 ^oC, 3 h; (b) Cl-POCEN^{i}Pr$_2$, DIPEA, DCM, 2 h, rt. (c) HATU, TEA, DMF, rt; (d) Ac_2O, DMAP, DCM, 1 h; (e) TBAF, THF, rt; (f) *N*-Hydroxysuccinimide, EDCI, DCM, overnight, rt. (g) MsCl, TEA, DCM, rt; (h) NaN_3, MeOH/H_2O, 70 ^oC.

Scheme S2. Diagram for the synthesis of modified reverse strand

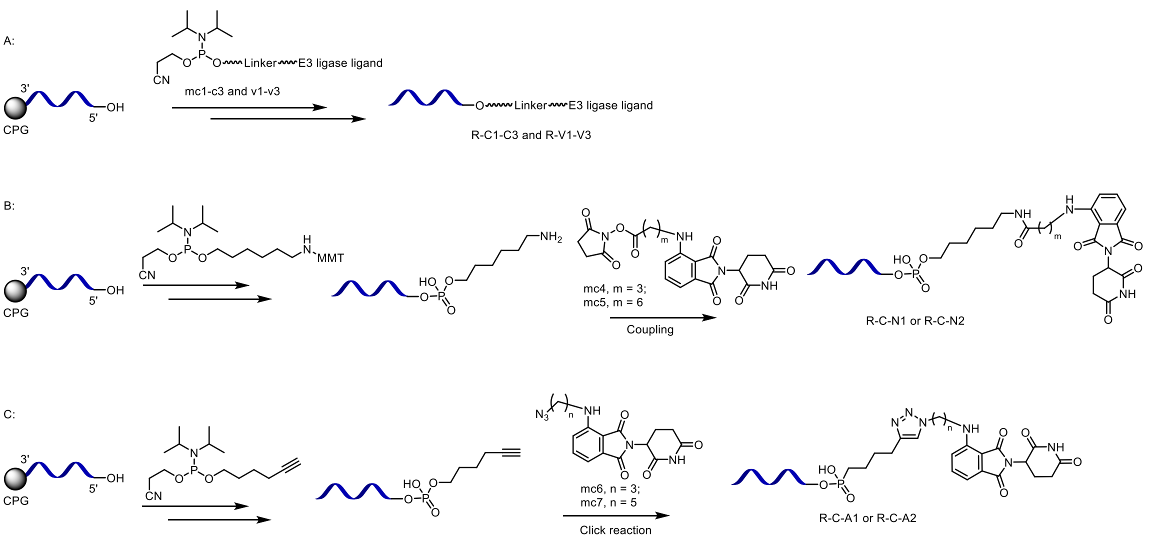

Materials and Methods

1. Chemical synthesis

4-Fluoro-thalidomide and VH 032 amine were prepared according to literature procedures [1].

Synthesis of compound 2a-c: Compound 4-fluoro-thalidomide (1.0 equiv) was dissolved in NMP, DIPEA (2.0 equiv) and 1a-c (1.5 equiv) were added, the mixture was heated to 100 ^{o}C under microwave condition for 3 hours. Then the mixture was absorbed on diatomite and purified by reversed-phase flash chromatography (H_2O: MeOH=90:10 to 50:50), giving compounds **2a-c**.

2-(2,6-dioxopiperidin-3-yl)-4-((5-hydroxypentyl)amino)isoindoline-1,3-dione (2a): Yellow solid, 65%.^{1}H NMR (400 MHz, $CDCl_3$) δ 8.16 (s, 1H), 7.49 (dd, *J* = 8.5, 7.1 Hz, 1H), 7.09 (d, *J* = 7.1 Hz, 1H), 6.88 (d, *J* = 8.5 Hz, 1H), 4.91 (dd, *J* = 12.1, 5.4 Hz, 1H), 3.66 (q, *J* = 6.3 Hz, 2H), 3.28 (t, *J* = 7.0 Hz, 2H), 2.93 – 2.67 (m, 3H), 2.12 (m, 1H), 1.75 – 1.66 (m, 2H), 1.64 – 1.59 (m, 2H), 1.54 – 1.46 (m, 2H).

2-(2,6-dioxopiperidin-3-yl)-4-((2-(2-(2-hydroxyethoxy)ethoxy)ethyl)amino)isoindoline-1,3-dione (2b): Yellow oil, 40%. ^{1}H NMR (400 MHz, $CDCl_3$) δ 8.31 (s, 1H), 7.48 (dd, *J* = 8.5, 7.2 Hz, 1H), 7.10 (d, *J* = 7.1 Hz, 1H), 6.90 (d, *J* = 8.5 Hz, 1H), 4.91 (dd, *J* = 11.9, 5.3 Hz, 1H), 3.76 – 3.70 (m, 4H), 3.69 – 3.64 (m, 4H), 3.62 – 3.58 (m, 2H), 3.47 (t, *J* = 5.3 Hz, 2H), 2.90 – 2.65 (m, 3H), 2.15 – 2.07 (m, 1H).

2-(2,6-dioxopiperidin-3-yl)-4-((2-(2-(2-(2-hydroxyethoxy)ethoxy)ethoxy)ethyl)amino)isoindoline-1,3-dione (2c): Yellow oil, 30%. ^{1}H NMR (400 MHz, DMSO-d_6) δ 11.08 (s, 1H), 7.63 – 7.55 (dd, *J* = 8.5, 7.0 Hz, 1H), 7.15 (d, *J* = 8.5 Hz, 1H), 7.04 (d, *J* = 7.0 Hz, 1H), 6.60 (t, *J* = 5.9 Hz, 1H), 5.05 (dd, *J* = 13.0, 5.4 Hz, 1H), 4.55 (t, *J* = 5.4 Hz, 1H), 3.62 (t, *J* = 5.3 Hz, 2H), 3.59 – 3.43 (m, 12H), 3.39 (t, *J* = 5.2 Hz, 2H), 2.94 – 2.82 (m, 1H), 2.56 (m, 2H), 2.08 – 1.96 (m, 1H).

***Synthesis of compound mc1-3*:** compound 2a-c (1.0 equiv) was dissolved in anhydrous DCM, DIPEA (2.0 equiv) and Cl-POCEN$^{i}Pr_2$ (1.5 equiv) was added. The mixture was stirred at room temperature for 1 hour. Solvent was removed, and the residue was purified with flash chromatography (Hexane:Actone

(5%TEA)=100:0 to75:25), giving product **mc1-3**.

2-cyanoethyl (5-((2-(2,6-dioxopiperidin-3-yl)-1,3-dioxoisoindolin-4-yl)amino)pentyl) diisopropyl phosphoramidite (mc1): Yellow oil, 65%. ^{1}H NMR (400 MHz, DMSO-d_6) δ 11.08 (s, 1H), 7.57 (t, *J* = 7.9 Hz, 1H), 7.09 (d, *J* = 8.5 Hz, 1H), 7.01 (d, *J* = 6.2 Hz, 1H), 6.54 (s, 1H), 5.04 (dd, *J* = 12.4, 4.5 Hz, 1H), 3.78 – 3.65 (m, 2H), 3.64 – 3.45 (m, 4H), 3.29 (m, 2H), 2.95 – 2.82 (m, 1H), 2.74 (t, *J* = 5.4 Hz, 2H), 2.63 – 2.52 (m, 2H), 2.02 (d, *J* = 12.2 Hz, 1H), 1.59 (m, 4H), 1.42 (m, 2H), 1.15 (d, *J* = 7.3 Hz, 12H).

2-cyanoethyl (2-(2-(2-((2-(2,6-dioxopiperidin-3-yl)-1,3-dioxoisoindolin-4-yl)amino)ethoxy)ethoxy) ethyl) diisopropylphosphoramidite (mc2): Yellow oil, 68%. ^{1}H NMR (400 MHz, DMSO-d_6) δ 11.08 (s, 1H), 7.61 – 7.54 (dd, *J* = 8.6, 7.1 Hz, 1H), 7.14 (d, *J* = 8.6 Hz, 1H), 7.04 (d, *J* = 7.1 Hz, 1H), 6.60 (t, *J* = 5.7 Hz, 1H), 5.05 (dd, *J* = 12.9, 5.4 Hz, 1H), 3.79 – 3.66 (m, 2H), 3.61 (m, 2H), 3.59 – 3.50 (m, 10H), 3.47 (m, 2H), 2.88 (m, 1H), 2.75 (t, *J* = 6.0 Hz, 2H), 2.63 – 2.52 (m, 2H), 2.06 – 1.99 (m, 1H), 1.12 (d, *J* = 6.7, 12H).

2-cyanoethyl (2-(2-(2-(2-((2-(2,6-dioxopiperidin-3-yl)-1,3-dioxoisoindolin-4-yl)amino)ethoxy)ethoxy) ethoxy)ethyl) diisopropylphosphoramidite (mc3): Yellow oil, 48%. ^{1}H NMR (400 MHz, DMSO-d_6) δ 11.08 (s, 1H), 7.58 (dd, *J* = 8.5, 7.2 Hz, 1H), 7.14 (d, *J* = 8.6 Hz, 1H), 7.04 (d, *J* = 7.0 Hz, 1H), 6.60 (t, *J* = 5.7 Hz, 1H), 5.05 (dd, *J* = 12.9, 5.4 Hz, 1H), 4.03 (m, 2H), 3.76 – 3.67 (m, 3H), 3.66 – 3.59 (m, 3H), 3.59 – 3.50 (m, 8H), 3.50 – 3.37 (m, 4H), 2.94 – 2.82 (m, 1H), 2.75 (t, *J* = 6.0 Hz, 2H), 2.63 – 2.53 (m, 2H), 2.06 – 1.98 (m, 1H), 1.15 – 1.07 (m, 12H).

***Synthesis of compound 2d-f*:** Compound VH 032 (1.0 equiv) was dissolved in DCM and DMF (1:1), and TEA (3.0 equiv), **1d-f** (1.5 equiv), and HATU (1.5 equiv) was added. The mixture was stirred at rt overnight. The reaction solution was diluted with DCM, washed with $NaHCO_3$ solution. The organic phase was concentrated and purified with flash chromatography (DCM:MeOH = 100:0 to 98:2), giving compound **2d-f**.

(2S,4R)-1-((S)-2-(6-((tert-butyldiphenylsilyl)oxy)hexanamido)-3,3-dimethylbutanoyl)-4-hydroxy-N-(4-(

4-methylthiazol-5-yl)benzyl)pyrrolidine-2-carboxamide (2d) : White foam solid, 70%. ^{1}H NMR (400 MHz, $CDCl_3$) δ 8.72 (s, 1H), 7.64 (m, 4H), 7.44 – 7.32 (m, 11H), 6.08 (d, *J* = 8.7 Hz, 1H), 4.70 (t, *J* = 7.9 Hz, 1H), 4.56 (dd, *J* = 15.0, 6.6 Hz, 1H), 4.49 (d, *J* = 8.8 Hz, 2H), 4.33 (dd, *J* = 15.0, 5.2 Hz, 1H), 4.11 – 4.05 (m, 1H), 3.61 (m, 3H), 2.57 – 2.49 (m, 4H), 2.16 (t, *J* = 7.6 Hz, 2H), 2.13 – 2.03 (m, 1H), 1.63 – 1.50 (m, 4H), 1.41 – 1.30 (m, 2H), 1.05 – 1.00 (s, 9H), 0.92 (s, 9H).

(2S,4R)-1-((S)-14-(tert-butyl)-2,2-dimethyl-12-oxo-3,3-diphenyl-4,7,10-trioxa-13-aza-3-silapentadecan-15-oyl)-4-hydroxy-N-(4-(4-methylthiazol-5-yl)benzyl)pyrrolidine-2-carboxamide (2e): Colorless oil, 62%. ^{1}H NMR (400 MHz, $CDCl_3$) δ 8.72 (s, 1H), 7.69 – 7.63 (m, 4H), 7.44 – 7.28 (m, 12H), 4.73 (t, *J* = 7.9 Hz, 1H), 4.54 (m, 2H), 4.43 (d, *J* = 8.3 Hz, 1H), 4.32 (dd, *J* = 15.0, 5.3 Hz, 1H), 4.12 (d, *J* = 11.4 Hz, 1H), 3.99 (q, *J* = 15.8 Hz, 2H), 3.80 (dd, *J* = 7.8, 3.3 Hz, 2H), 3.71 – 3.54 (m, 7H), 2.56 (m, 1H), 2.51 (s, 3H), 2.14 – 2.06 (m, 1H), 1.06 – 1.00 (s, 9H), 0.92 (s,9H).

(2S,4R)-1-((S)-17-(tert-butyl)-2,2-dimethyl-15-oxo-3,3-diphenyl-4,7,10,13-tetraoxa-16-aza-3-silaoctade can-18-oyl)-4-hydroxy-N-(4-(4-methylthiazol-5-yl)benzyl)pyrrolidine-2-carboxamide (2f): Colorless oil, 60%. ^{1}H NMR (400 MHz, $CDCl_3$) δ 8.70 (s, 1H), 7.69 – 7.64 (m, 4H), 7.43 – 7.32 (m, 12H), 4.72 (t, *J* = 7.9 Hz, 1H), 4.53 (m, 2H), 4.47 (d, *J* = 8.5 Hz, 1H), 4.33 (dd, *J* = 15.0, 5.3 Hz, 1H), 4.08 (d, *J* = 10.2 Hz, 1H), 4.03 – 3.91 (m, 2H), 3.79 (t, *J* = 5.3 Hz, 2H), 3.68 – 3.55 (m, 11H), 2.56 – 2.48 (m, 4H), 2.15 – 2.06 (m, 1H), 1.03 (s, 9H), 0.94 (s, 9H).

***Synthesis of compound 3a-c*:** Compound **2d-f** (1.0 equiv) was dissolved in DCM and cooled to 0 ^{o}C, then TEA (1.5 equiv) and DMAP (0.01 equiv) was added. The mixture was stirred and Ac_2O (1.5 equiv) was added slowly. The reaction was stirred at 0°C for 1h. the reaction solution was washed with water, and the organic phase was dried with Na_2SO_4, filtered and concentrated. The residue was purified with flash chromatography (DCM:MeOH = 100:0 to 98:2), giving compound **3a-c**.

(3R,5S)-1-((S)-2-(6-((tert-butyldiphenylsilyl)oxy)hexanamido)-3,3-dimethylbutanoyl)-5-((4-(4-methylthiazol-5-yl)benzyl)carbamoyl)pyrrolidin-3-yl acetate (3a): White foam solid, 90%. ^{1}H NMR (400 MHz, CDCl$_3$) ^{1}H NMR (400 MHz, cdcl$_3$) δ 8.89 (d, *J* = 3.7 Hz, 1H), 7.64 (dd, *J* = 7.6, 1.3 Hz, 4H), 7.43 – 7.32 (m, 10H), 7.18 – 7.13 (m, 1H), 6.04 (d, *J* = 9.1 Hz, 1H), 5.37 (s, 1H), 4.70 – 4.65 (m, 1H), 4.62 – 4.50 (m, 2H), 4.34 (dd, *J* = 14.9, 5.3 Hz, 1H), 4.05 (d, *J* = 12.7 Hz, 1H), 3.84 – 3.76 (m, 1H), 3.63 (t, *J* = 6.4 Hz, 2H), 2.71 (m, 1H), 2.54 (s, 3H), 2.17 (m, 3H), 2.03 (s, 3H), 1.57 (m, 4H), 1.36 (m, 2H),1.03 (s, 9H), 0.89 (s, 9H).

(3R,5S)-1-((S)-14-(tert-butyl)-2,2-dimethyl-12-oxo-3,3-diphenyl-4,7,10-trioxa-13-aza-3-silapentadecan-15-oyl)-5-((4-(4-methylthiazol-5-yl)benzyl)carbamoyl)pyrrolidin-3-yl acetate (3b): Colorless oil, 92%. ^{1}H NMR (400 MHz, CDCl$_3$) δ 8.76 (s, 1H), 7.66 (dd, *J* = 7.8, 1.5 Hz, 4H), 7.43 – 7.30 (m, 11H), 7.22 (d, *J* = 8.4 Hz, 1H), 5.36 (s, 1H), 4.73 – 4.67 (m, 1H), 4.56 – 4.47 (m, 2H), 4.33 (dd, *J* = 14.9, 5.4 Hz, 1H), 4.05 (d, *J* = 11.9 Hz, 1H), 3.99 (m, 2H), 3.84 – 3.75 (m, 3H), 3.70 – 3.56 (m, 6H), 2.77 – 2.69 (m, 1H), 2.52 (s, 3H), 2.15 (m, 1H), 2.03 (s, 3H), 1.03 (s, 9H), 0.90 (s, 9H).

(3R,5S)-1-((S)-17-(tert-butyl)-2,2-dimethyl-15-oxo-3,3-diphenyl-4,7,10,13-tetraoxa-16-aza-3-silaoctadecan-18-oyl)-5-((4-(4-methylthiazol-5-yl)benzyl)carbamoyl)pyrrolidin-3-yl acetate (3c): Colorless oil, 87%. ^{1}H NMR (400 MHz, CDCl$_3$) δ 8.75 (s, 1H), 7.69 – 7.64 (m, 4H), 7.43 – 7.31 (m, 11H), 7.23 (d, 7.4 Hz, 1H), 5.36 (s, 1H), 4.71 (dd, *J* = 8.2, 6.6 Hz, 1H), 4.57 – 4.49 (m, 2H), 4.34 (dd, *J* = 14.9, 5.4 Hz, 1H), 4.05 (d, *J* = 13.7 Hz, 1H), 3.98 (m, 2H), 3.80 (m, 3H), 3.70 – 3.61 (m, 8H), 3.57 (t, *J* = 5.3 Hz, 2H), 2.77 – 2.68 (m, 1H), 2.52 (s, 3H), 2.20 – 2.13 (m, 1H), 2.04 (s, 3H), 1.06 – 1.01 (s, 9H), 0.91 (s, 9H).

***Synthesis of compound 4a-c*:** Compound 3a-c (1.0 equiv) was dissolved in THF and TBAF (1M in THF, 2.0 equiv) was added. The mixture was stirred at rt overnight. The solvent was removed and the residue was purified with flash chromatography (DCM:MeOH = 100:0 to 97:3), giving compound 4a-c.

(3R,5S)-1-((S)-2-(6-hydroxyhexanamido)-3,3-dimethylbutanoyl)-5-((4-(4-methylthiazol-5-yl)benzyl)car

bamoyl)pyrrolidin-3-yl acetate (4a): White solid, 60%. ^{1}H NMR (400 MHz, $CDCl_3$) δ 8.75 (s, 1H), 7.40 – 7.32 (m, 4H), 7.20 (t, *J* = 6.0 Hz, 1H), 6.03 (d, *J* = 9.2 Hz, 1H), 5.37 (m, 1H), 4.73 – 4.65 (m, 1H), 4.57 (dd, *J* = 14.9, 6.6 Hz, 1H), 4.51 (d, *J* = 9.2 Hz, 1H), 4.34 (dd, *J* = 14.9, 5.2 Hz, 1H), 4.07 (d, *J* = 11.7 Hz, 1H), 3.79 (dd, *J* = 11.6, 4.6 Hz, 1H), 3.66 – 3.57 (m, 2H), 2.75 – 2.66 (m, 1H), 2.54 (s, 3H), 2.19 (m, 3H), 2.05 (s, 3H), 1.64 (m, 2H), 1.60 – 1.51 (m, 2H), 1.47 (m, 2H), 0.90 (s, 9H).

(3R,5S)-1-((S)-2-(2-(2-(2-hydroxyethoxy)ethoxy)acetamido)-3,3-dimethylbutanoyl)-5-((4-(4-methylthiazol-5-yl)benzyl)carbamoyl)pyrrolidin-3-yl acetate (4b): White solid, 68%. ^{1}H NMR (400 MHz, $CDCl_3$) δ 8.72 (s, 1H), 7.54 (d, *J* = 9.5 Hz, 1H), 7.37 (s, 4H), 7.16 (t, *J* = 5.8 Hz, 1H), 5.40 (m, 1H), 4.66 (dd, *J* = 8.2, 6.7 Hz, 2H), 4.57 (dd, *J* = 14.8, 6.6 Hz, 1H), 4.34 (dd, *J* = 14.8, 5.4 Hz, 1H), 4.05 (dd, *J* = 16.1, 5.5 Hz, 1H), 3.98 – 3.90 (m, 2H), 3.83 (dd, *J* = 11.8, 4.7 Hz, 1H), 3.78 – 3.56 (m, 9H), 2.75 – 2.67 (m, 1H), 2.53 (d, *J* = 3.3 Hz, 3H), 2.18 (m, 1H), 2.04 (d, *J* = 2.5 Hz, 3H), 0.92 (s, 9H).

(3R,5S)-1-((S)-2-(tert-butyl)-14-hydroxy-4-oxo-6,9,12-trioxa-3-azatetradecanoyl)-5-((4-(4-methylthiazol-5-yl)benzyl)carbamoyl)pyrrolidin-3-yl acetate (4c): Colorless oil, 52%. ^{1}H NMR (400 MHz, $CDCl_3$) δ 8.72 (s, 1H), 7.50 (m, 1H), 7.39 – 7.28 (m, 5H), 5.39 (m, 1H), 4.69 (m, 1H), 4.57 (m, 2H), 4.33 (dd, *J* = 14.9, 5.3 Hz, 1H), 4.04 – 3.99 (m, 3H), 3.84 (dd, *J* = 11.6, 4.9 Hz, 1H), 3.72 – 3.55 (m, 12H), 2.74 – 2.65 (m, 1H), 2.53 (s, 3H), 2.21 – 2.12 (m, 1H), 2.04 (s, 3H), 0.93 (s, 9H).***Synthesis of compound mv1-3*:** compound 4a-c (1.0 equiv) was dissolved in anhydrous DCM, DIPEA (2.0 equiv) and Cl-POCEN^iPr_2 (1.5 equiv) was added. The mixture was stirred at room temperature for 1 hour. Solvent was removed, and the residue was purified with flash chromatography (Hexane:Actone (5%TEA)=100:0 to 60:40), giving product as colorless oil.

(3R,5S)-1-((2S)-2-(6-(((2-cyanoethoxy)(diisopropylamino)phosphaneyl)oxy)hexanamido)-3,3-dimethylbutanoyl)-5-((4-(4-methylthiazol-5-yl)benzyl)carbamoyl)pyrrolidin-3-yl acetate (mv1): Colorless oil, 60%. ^{1}H NMR (400 MHz, $CDCl_3$) δ 8.68 (s, 1H), 7.36 (m, 4H), 7.19 (t, *J* = 5.7 Hz, 1H), 6.01 (d, *J* = 9.1 Hz,

1H), 5.37 (s, 1H), 4.71 (t, *J* = 7.4 Hz, 1H), 4.60 – 4.50 (m, 2H), 4.34 (dd, *J* = 14.7, 5.1 Hz, 1H), 4.05 (d, *J* = 11.4 Hz, 1H), 3.88 – 3.73 (m, 3H), 3.68 – 3.53 (m, 4H), 2.79 – 2.72 (m, 1H), 2.63 (t, *J* = 6.5 Hz, 2H), 2.52 (s, 3H), 2.25 – 2.12 (m, 3H), 2.05 (s, 3H), 1.61 (m, 4H), 1.48 – 1.34 (m, 2H), 1.16 (m, 12H), 0.89 (s, 9H).

(3R,5S)-1-((2S)-2-(2-(2-(2-(((2-yanoethoxy)(diisopropylamino)phosphaneyl)oxy)ethoxy)ethoxy) acetamido)-3,3-dimethylbutanoyl)-5-((4-(4-methylthiazol-5-yl)benzyl)carbamoyl)pyrrolidin-3-yl acetate (mv2): Colorless oil, 67%. ^{1}H NMR (400 MHz, $CDCl_3$) δ 8.67 (s, 1H), 7.36 (q, *J* = 8.2 Hz, 4H), 7.26 – 7.22 (m, 1H), 7.19 (d, *J* = 9.2 Hz, 1H), 5.37 (m, 1H), 4.72 (dd, *J* = 8.0, 6.7 Hz, 1H), 4.59 – 4.48 (m, 2H), 4.35 (dd, *J* = 14.9, 5.3 Hz, 1H), 4.07 – 4.02 (m, 1H), 4.00 (d, *J* = 3.5 Hz, 2H), 3.91 – 3.76 (m, 4H), 3.75 – 3.64 (m, 7H), 3.59 (m, 2H), 2.79 – 2.70 (m, 1H), 2.66 – 2.61 (m, 2H), 2.52 (s, 3H), 2.21 – 2.12 (m, 1H), 2.04 (s, 3H), 1.19 – 1.14 (m, 12H), 0.91 (s, 9H).

(3R,5S)-1-((2S)-2-(tert-butyl)-14-(((2-cyanoethoxy)(diisopropylamino)phosphaneyl)oxy)-4-oxo-6,9,12-trioxa-3-azatetradecanoyl)-5-((4-(4-methylthiazol-5-yl)benzyl)carbamoyl)pyrrolidin-3-yl acetate (mv3): Colorless oil, 40%. ^{1}H NMR (400 MHz, $CDCl_3$) δ 8.68 (s, 1H), 7.36 (q, *J* = 8.1 Hz, 4H), 7.25 – 7.17 (m, 2H), 5.37 (m, 1H), 4.75 – 4.69 (m, 1H), 4.59 – 4.49 (m, 2H), 4.36 (dd, *J* = 14.9, 5.3 Hz, 1H), 4.07 – 4.02 (m, 1H), 4.00 (d, *J* = 4.7 Hz, 2H), 3.90 – 3.75 (m, 4H), 3.75 – 3.53 (m, 13H), 2.80 – 2.71 (m, 1H), 2.64 (t, *J* = 6.5 Hz, 2H), 2.52 (s, 3H), 2.16 (m, 1H), 2.04 (s, 3H), 1.21 – 1.14 (m, 12H), 0.92 (s, 9H).

Synthesis of compound 5a-c: Compound 4-fluoro-thalidomide (1.0 equiv) was dissolved in DMA, DIPEA (2.0 equiv) and compound 1g-i (1.5 equiv) were added, the mixture was heated to 100 ^{o}C in sealed tube overnight. then the mixture was concentrated and purified by reverse phase flash chromatography (H_2O: MeOH=100:0 to 50:50), giving compounds **5a-c**.

4-((2-(2,6-dioxopiperidin-3-yl)-1,3-dioxoisoindolin-4-yl)amino)butanoic acid (5a): ^{1}H NMR (400 MHz, DMSO-*d*$_6$) δ 11.10 (s, 1H), 7.58 (t, *J* = 7.8 Hz, 1H), 7.13 (d, *J* = 8.6 Hz, 1H), 7.02 (d, *J* = 7.1 Hz, 1H), 6.66 (t,

J = 5.8 Hz, 1H), 5.05 (dd, *J* = 12.8, 5.1 Hz, 1H), 3.31 (m, 2H), 2.94 – 2.81 (m, 1H), 2.64 – 2.51 (m, 2H), 2.30 (t, *J* = 7.1 Hz, 2H), 2.02 (d, *J* = 6.8 Hz, 1H), 1.78 (m, 2H).

7-((2-(2,6-dioxopiperidin-3-yl)-1,3-dioxoisoindolin-4-yl)amino)heptanoic acid (5b): ^{1}H NMR (400 MHz, DMSO-d_6) δ 12.00 (s, 1H), 11.10 (s, 1H), 7.58 (t, *J* = 7.8 Hz, 1H), 7.09 (d, *J* = 8.6 Hz, 1H), 7.02 (d, *J* = 7.0 Hz, 1H), 6.54 (t, *J* = 5.7 Hz, 1H), 5.05 (dd, *J* = 12.9, 5.2 Hz, 1H), 3.31 – 3.24 (m, 2H), 2.88 (m, 1H), 2.55 (m, 2H), 2.20 (t, *J* = 7.3 Hz, 2H), 2.07 – 1.97 (m, 1H), 1.61 – 1.44 (m, 4H), 1.32 (m, 4H).

2-(2,6-dioxopiperidin-3-yl)-4-((3-hydroxypropyl)amino)isoindoline-1,3-dione (5c): Yellow solid, 60%. ^{1}HNMR (400 MHz, $CDCl_3$) δ 8.15 (s, 1H), 7.50 (t, *J* = 7.8 Hz, 1H), 7.09 (d, *J* = 7.1 Hz, 1H), 6.93 (d, *J* = 8.5 Hz, 1H), 4.92 (dd, *J* = 11.9, 5.1 Hz, 1H), 3.82 (t, *J* = 5.7 Hz, 2H), 3.44 (t, *J* = 6.6 Hz, 2H), 2.93 – 2.66 (m, 3H), 2.16 – 2.07 (m, 1H), 1.96 – 1.87 (m, 2H).

Synthesis of compound mc4-5: Compound 5a or 5b (1.0 equiv) and *N*-Hydroxysuccinimide (1.5 equiv) were mixed in DCM, cool to 0°C, then EDCI (1.3 equiv) was added slowly. The mixture was stirred at RT overnight. The reaction was diluted with DCM and washed, with H_2O and brine. The organic phase was dried with Na_2SO_4, filtered and concentrated, giving **mc4-5** as yellow solid.

2,5-dioxopyrrolidin-1-yl 4-((2-(2,6-dioxopiperidin-3-yl)-1,3-dioxoisoindolin-4-yl)amino)butanoate (mc4): 88%; LC-MS (ESI$^+$): m/z 457.2 [M + H$^+$]

2,5-dioxopyrrolidin-1-yl 7-((2-(2,6-dioxopiperidin-3-yl)-1,3-dioxoisoindolin-4-yl)amino)heptanoate (mc5): 85%; LC-MS (ESI$^+$): m/z 499.3 [M + H$^+$]

Synthesis of compound mc6-7: Compound 5c or 2 was dissolved in DCM, TEA (2.0 equiv) and MsCl (1.2 equiv) were added, the mixture was stirred at RT for 2h. The reaction was added water, then extracted with DCM, the organic phase was dried and concentrated. The residue was dissolved in DCM MeOH/H_2O and NaN_3 was added, then the mixture was heated to 70 ^{o}C overnight. Solvent was removed, to the residue was

added water, then extracted with EA twice. The organic phase was concentrated and purified by flash chromatography (DCM: EA=100:0 to 85:15), giving compounds **mc6-7**.

4-((3-azidopropyl)amino)-2-(2,6-dioxopiperidin-3-yl)isoindoline-1,3-dione (mc6): Yellow solid, 30%. ^{1}H NMR (400 MHz, $CDCl_3$) δ 8.07 (s, 1H), 7.52 (t, *J* = 7.8 Hz, 1H), 7.12 (d, *J* = 7.1 Hz, 1H), 6.92 (d, *J* = 8.6 Hz, 1H), 6.29 (s, 1H), 4.92 (dd, *J* = 11.9, 5.2 Hz, 1H), 3.47 (t, *J* = 6.3 Hz, 2H), 3.41 (t, *J* = 6.7 Hz, 2H), 2.80 (m, 3H), 2.19 – 2.08 (m, 1H), 1.92 (m, 2H).

4-((5-azidopentyl)amino)-2-(2,6-dioxopiperidin-3-yl)isoindoline-1,3-dione (mc7): Yellow solid, 46%. ^{1}H NMR (400 MHz, $CDCl_3$) δ 8.17 (s, 1H), 7.50 (t, *J* = 7.8 Hz, 1H), 7.09 (d, *J* = 7.1 Hz, 1H), 6.88 (d, *J* = 8.5 Hz, 1H), 6.24 (s, 1H), 4.91 (dd, *J* = 12.0, 5.3 Hz, 1H), 3.30 (m, 4H), 2.93 – 2.67 (m, 3H), 2.17 – 2.08 (m, 1H), 1.68 (m, 4H), 1.50 (m, 2H).

2. Synthesis of oligonucleotides and annealing reaction

All oligonucleotides used in this study were synthesized by ExonanoRNA (Columbus, OH). For oligo annealing reaction, single-stranded forward and reverse oligonucleotides were mixed in an assembly buffer (10 mM Tris-HCl [pH7.5], 100 mM NaCl, 1 mM EDTA), and heated to 90 ^oC for 5 min, then slowly cooled down to 37 ^oC within 1 h. Double-stranded O’PROTACs were mixed well, aliquoted and stored at -20 ^oC for the future use.

3. Cell culture and transfection

RWPE-1, C4-2, LNCaP, 22Rv1, VCaP, PC-3 and DU145 prostate cancer cell line and 293T cell line were purchased from the American Type Culture Collection (ATCC). BPH1 cell line was kindly provided by Dr. Simon Hayward at NorthShore University HealthSystem at Chigaco and LAPC4 cell line was kindly provided

by Dr. Charles Sawyers at Memorial Sloan Kettering Cancer Center at New York. 293T cells were maintained in DMEM medium with 10% FBS. RWPE-1 cells were cultured in keratinocyte serum free medium supplemented with 0.05 mg/ml bovine pituitary extract, 5 ng/ml epidermal growth factor, and 100 U/ml penicillin -100 µg/ml streptomycin mixture. VCaP cells were cultured in RPMI medium with 15% FBS. LAPC4 cells were cultured in IMEM with 10% FBS. All other cell lines were maintained in RPMI medium with 10% FBS. Cells were transiently transfected with O'PROTAC using Lipofectamine 2000 (Thermo Fisher) or polyethylenimine (PEI) (Cat#23966, Polysciences) according to the manufacturer's instructions.

4. Western blot

Cell lysate was subjected to SDS-PAGE and proteins were transferred to nitrocellulose membranes (GE Healthcare Sciences). The membranes were blocked in Tris-buffered saline (TBS, pH 7.4) containing 5% non-fat milk and 0.1% Tween-20, washed twice in TBS containing 0.1% Tween-20, and incubated with primary antibody overnight at 4 °C, followed by secondary antibody for 1 h at room temperature. The proteins of interest were visualized using ECL chemiluminescence system (Thermo Fisher).

5. Biotin pulldown assay

PC-3 cells were transfected with 100 nM of biotin-labelled LEF1 O' PROTACs OP-V1 to V3 using PEI (Polysciences) for 36 h. The cells were treated with MG132 for 12 h before lysed in lysis buffer containing 50 mM Tris-HCl (pH7.5), 150 mM NaCl, 1% NP-40, 0.5% sodium deoxycholate and 1% proteinase inhibitor. The cell lysate was incubated with Streptavidin Sepharose High Performance beads (GE Healthcare) overnight at 4 ^{o}C. The binding protein was eluted by elution buffer and subjected to western blot.

6. RNA extraction and RT-qPCR

RNA was extracted using TRIzol (Invitrogen) and reversely transcribed into cDNA with SuperScript III First-Strand Synthesis System (Promega). The quantitative PCR (qPCR) was performed in the iQ thermal cycler (Bio-Rad) using the iQ SYBR Green Supermix (Bio-Rad). Each sample was carried out in triplicate and three biological repeats were performed. The ΔCT was calculated by normalizing the threshold difference of a certain gene with glyceraldehyde-3-phosphate dehydrogenase (*GAPDH*). The primer sequences are listed as following:

CCND1	(F)GATCAAGTGTGACCCGGACT	(R)CTTGGGGTCCATGTTCTGCT
c-MYC	(F)TACAACACCCGAGCAAGGAC	(R)AGGCTGGTTTTCCACTACCC
LEF1	(F)AGCACGGAAAGAAAGACAGC	(R)TCTTGGACCTGTACCTGATGC
GAPDH	(F)TCGGAGTCAACGGATTTGGT	(R)TTCCCGTTCTCAGCCTTGAC

7. Immunofluorescent cytochemistry assay

PC-3 cells were seeded on the slides in 6-well plate overnight and reached to 60-70% of confluence and then transfected with LEF1 OP-V1 (0 nM or 100 nM). After 24 h, Cells were fixed by 4% paraformaldehyde and permeabilized with 0.05% Triton X-100. After 1h block at room temperature, cells were subjected to immunoblot with LEF1 antibody (#2230S, Cell Signaling Technology) at 4℃ overnight. After washing, cells were incubated with anti-rabbit Alexa Fluor® 594 (A-11012, Thermo Fishers) for 1 h at room temperature and mounted on the slides using the DAPI-containing counterstain solution (H-1200, Vector Laboratories) after washing. Images were taken by LSM 780 confocal microscope (Zeiss).

8. Cell growth assay

Cell viability was measured using the MTS assay according to the manufacture’s instruction (Promega).

PC-3 and DU145 cells were transfected with LEF1 OP-V1 for 48 h and 1,000 cells were seeded in each well of 96-well plates with 100 μL of medium. After cells adhered to the plate, at indicated time points, cell culture medium was replaced with 1 × PBS and 10 μL of CellTiter 96R Aqueous One Solution Reagent (Promega) was added to each well. The plates were incubated for 2 h at 37 °C in a cell incubator. Microplate reader was used to measure absorbance of 490 nm in each well.

9. Nuclear extraction and electrophoretic mobility shift assay (EMSA)

Nuclear protein was extracted using NE-PER™ Nuclear and Cytoplasmic Extraction Reagents (Cat# 78833, Thermo Fisher Scientific). EMSA was performed according to the manufacturer's instruction by using the biotin-labeled LEF1 or ERG OPROTAC as probes. For supershift assay, ERG or LEF1 antibodies were added into the cell nuclear extract mixed with the biotin-labelled OPROTAC probes and the mixture were incubated with for 1 h before loading into 6% of non-denatured polyacrylamide gel.

10. Three-dimensional (3D) culture

Twenty-thousands of VCaP cells were resuspended in 250 μl plain medium and seeded on the top of a thin layer of Matrigel Matrigel matrix (BD Bioscience) in a 24-well plate. After 30 min, when the cells were settled down, they were covered with a layer of 10% Matrigel diluted with DMEM/F12 medium. Cells were transfected with ERG OP-C-N1 (200 nM) and the medium was changed with fresh and warm DMEM/F12 plus 10% FBS medium every 2–3 days.

11. Mouse xenograft and drug treatment

The mouse experiments were approved by the Mayo Clinic Institutional Animal Care and Use Committee

(IACUC). 3 x 10^6 PC-3 cells or DU145 cells mixed with Matrigel matrix (BD Bioscience) were injected subcutaneously into the left flank of six-week-old SCID male mice. When the tumor volume reached approximately 75 mm^3, mice were randomly divided into three groups for treatment with 1 × PBS, control OP or LEF1 OP-V1 (10 mg/kg in PEI solution) via tail vein injection every other day. The volume of xenografts and mouse body weight were measured every three days. After 18-day (for PC-3 tumors) or 21-day (for DU145 tumors) treatment, mice were euthanized and xenografts were harvested for the measurement of weight. One part of tissues was formalin fixed and paraffin-embedded (FFPE) for IHC analysis and the rest of the tissues was used for RNA and protein extraction for RT-qPCR and Western blot analysis, respectively.

12. Immunohistochemistry (IHC)

The FFPE xenograft tissues were cut consecutively at 4 micrometer for the IHC assay. The IHC staining was performed as previously reported [2].

13. Statistical Analysis

Statistical analysis was performed with one-sided or two-sided paired Student’s t-test for single comparison. *P* value < 0.05 is considered statistically significant. All values shown are expressed as means ± SD.

Supplementary Figure 1

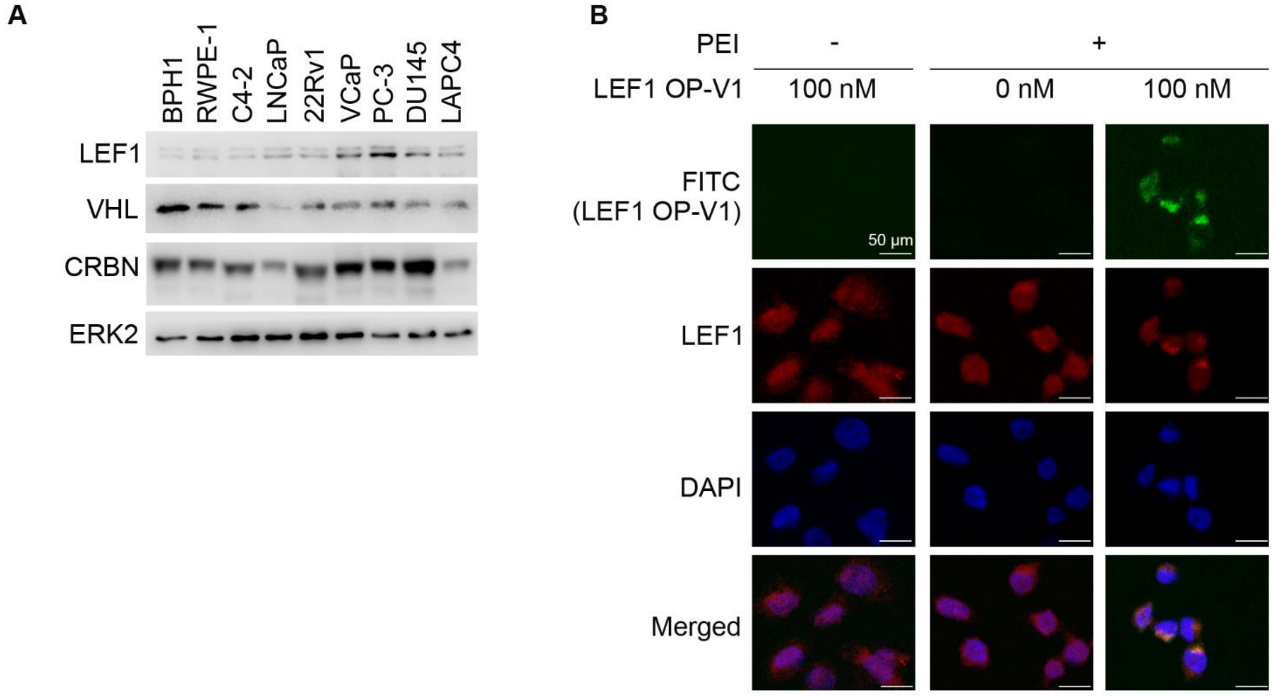

Figure S1. (A) Western blot analysis of expression of LEF1, VHL and CRBN protein in two immortalized, but not transformed prostatic cell lines (BPH1 and RWPE-1) and seven prostate cancer cell lines indicated. (B) Representative images of immunofluorescent cytochemistry showing the overlap between transfected FITC-labeled LEF1 OP-V1 (green) and the endogenous LEF1 protein (red) in the nuclei of PC-3 cells counterstained with DAPI.

Supplementary Figure 2

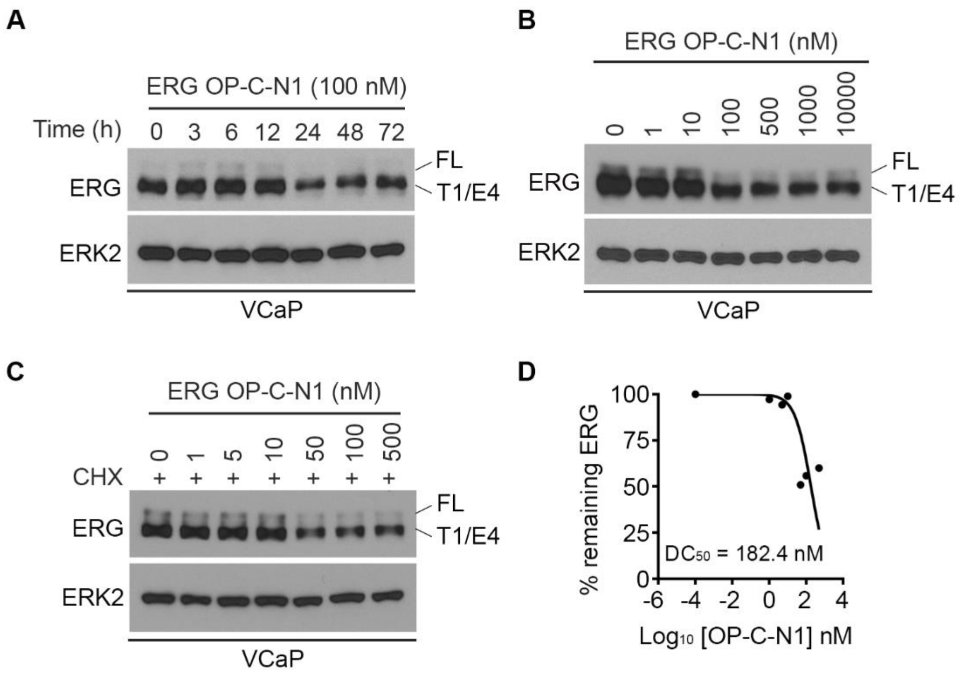

Figure S2. (A) VCaP cells were transfected with a final concentration of 100 nM and harvested at different time points, followed by western blot to detect ERG expression. (B) VCaP cells were transfected with increasing concentrations of ERG OP-C-N1 for 36 h, followed by western blot to detect ERG expression. (C and D) VCaP cells were transfected with increasing concentrations of ERG OP-C-N1 for 24 h and treated with 20 μg/ml cycloheximide (CHX) for another 12 h, followed by western blot to detect ERG expression (C). The remaining ERG protein (%) was calculated by normalizing the values to that in the group without ERG OP-C-N1 treatment, and DC_{50} was determined (D). This experiment was repeated once and similar results were obtained.

LEF1-F:

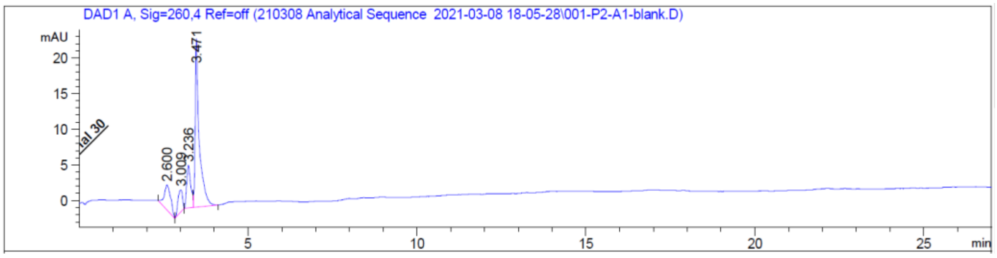

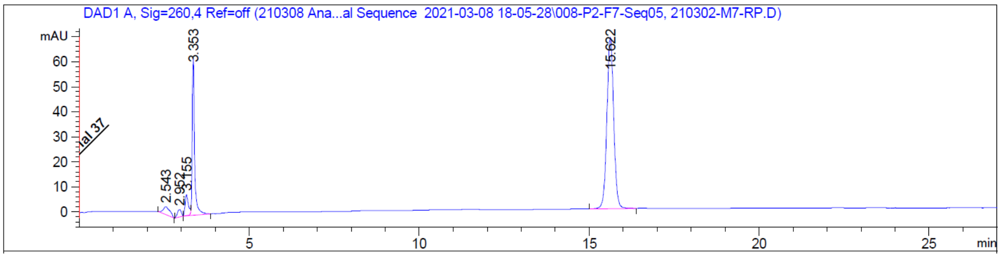

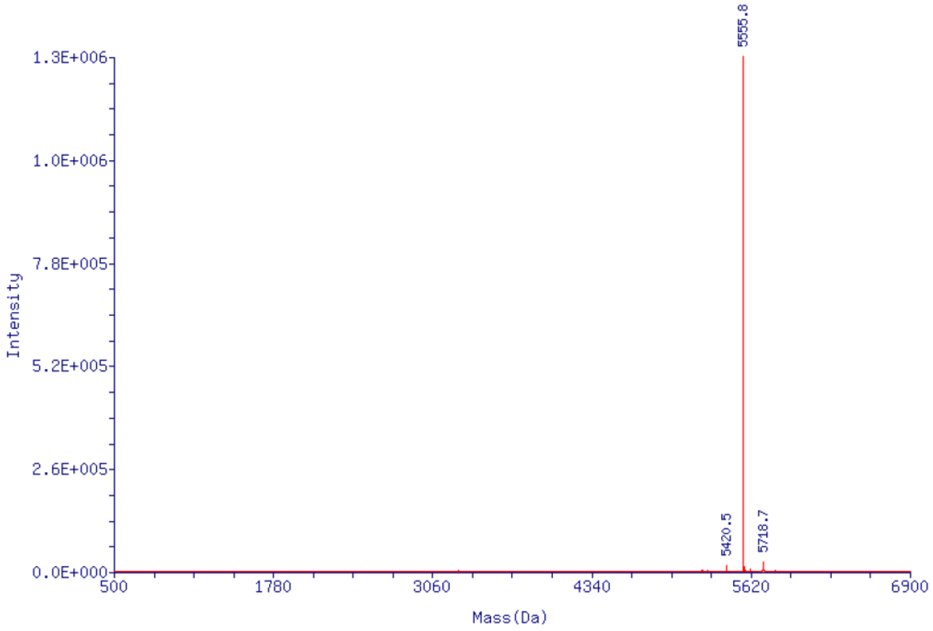

LEF1-F-Biotin:

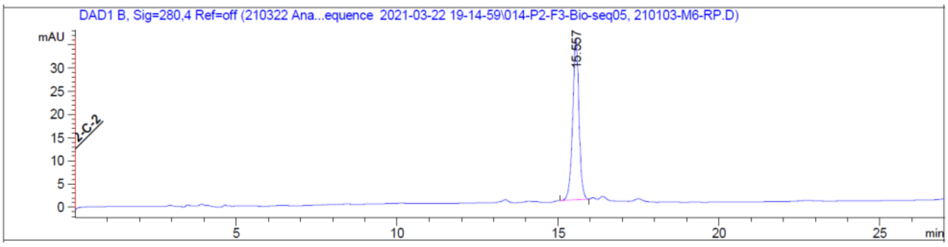

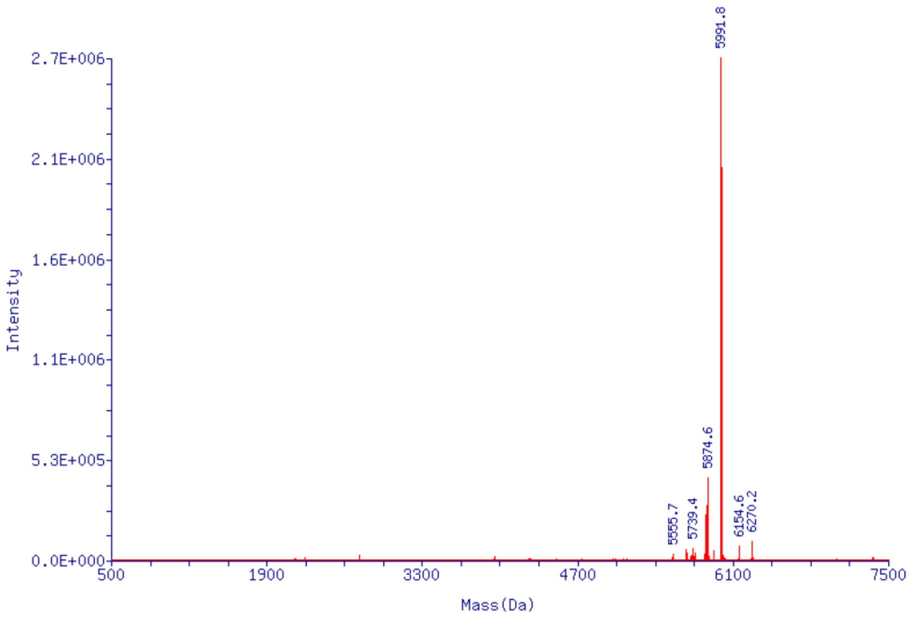

LEF1-F-FITC:

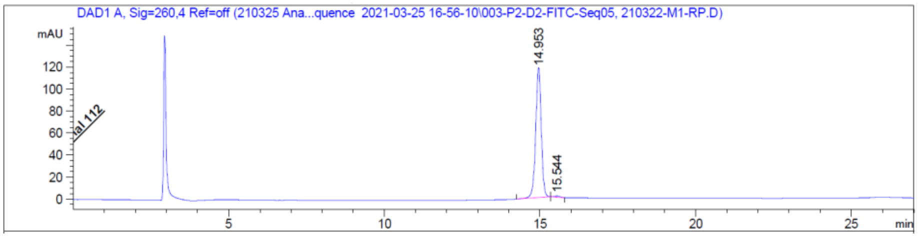

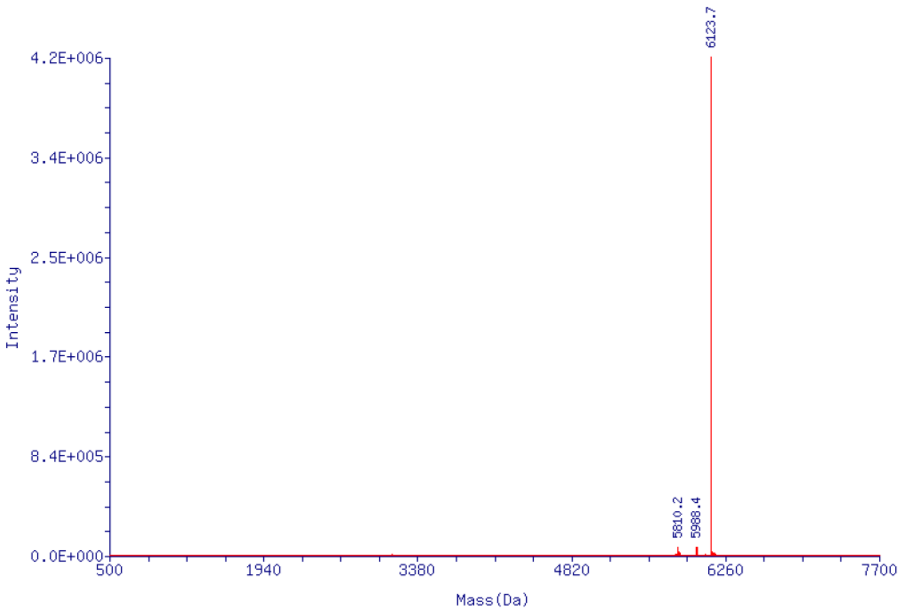

LEF1-R-V1:

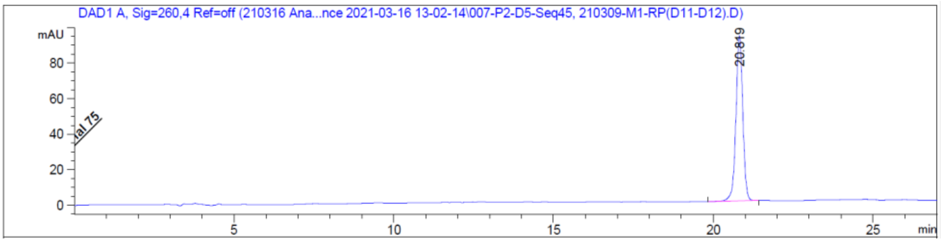

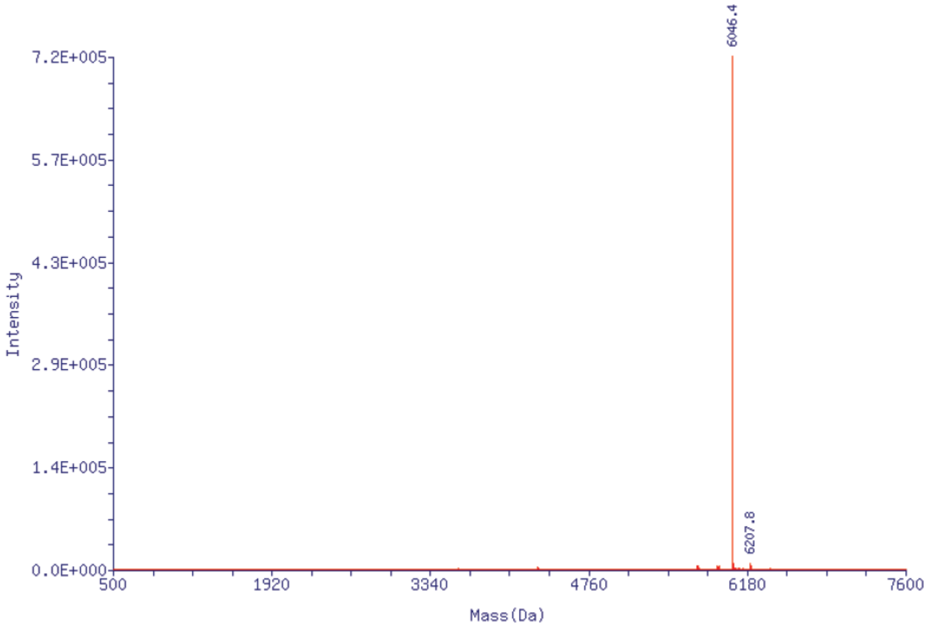

LEF1-R-V2:

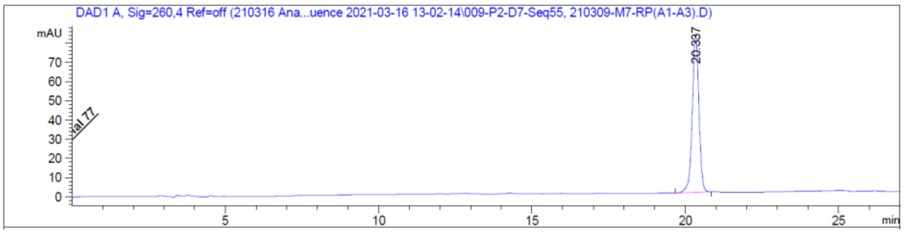

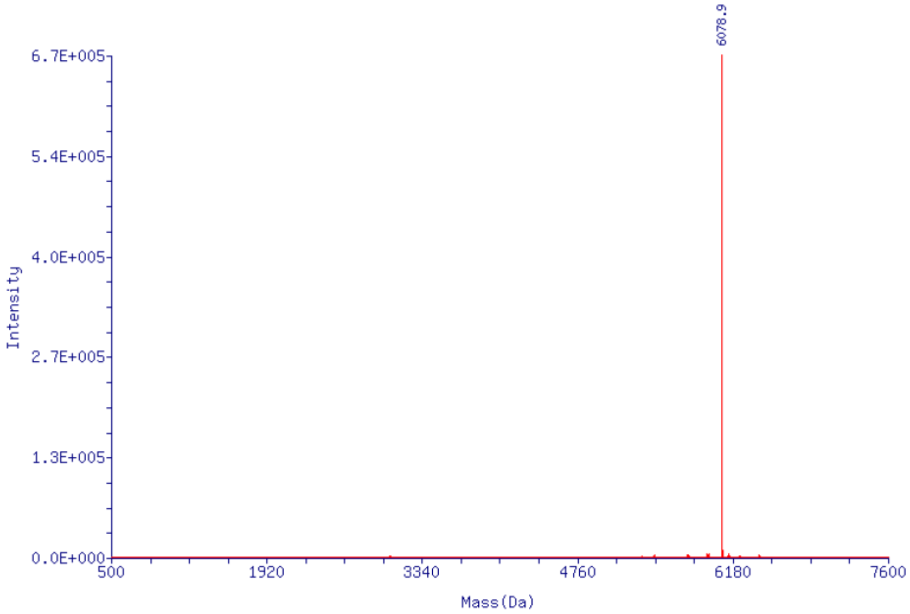

LEF1-R-V3:

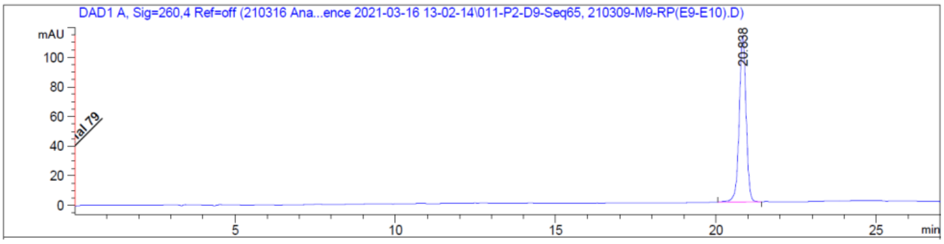
DAD1 A, Sig=260,4 Ref=off (210316 Ana...ence 2021-03-16 13-02-14\011-P2-D9-Seq65, 210309-M9-RP(E9-E10).D)
mAU
20.838
min

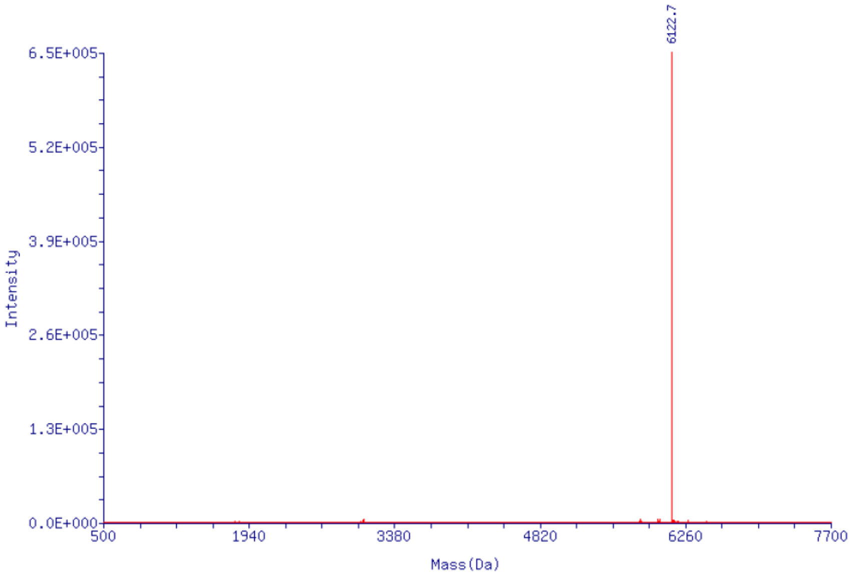
6122.7
Intensity
Mass(Da)

ERG-F:

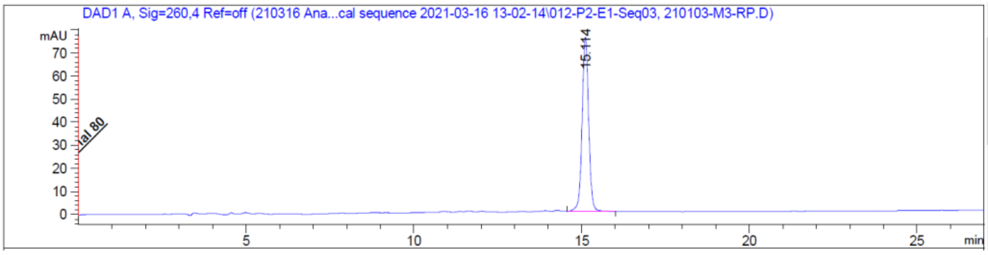
DAD1 A, Sig=260,4 Ref=off (210316 Ana...cal sequence 2021-03-16 13-02-14\012-P2-E1-Seq03, 210103-M3-RP.D)
mAU
15.114
min

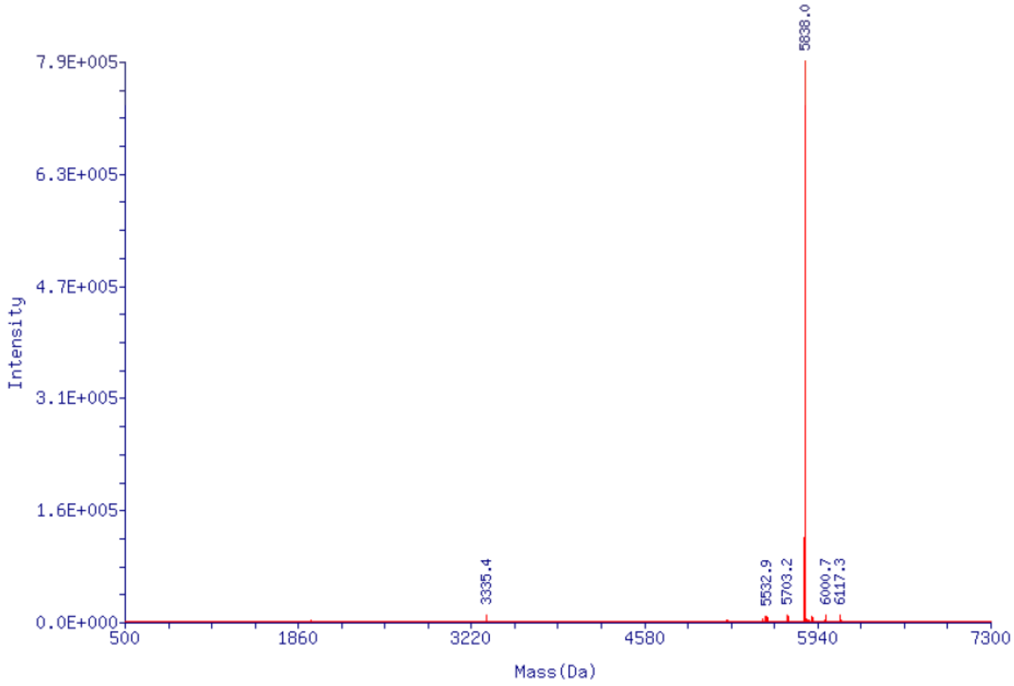

ERG-F-Biotin

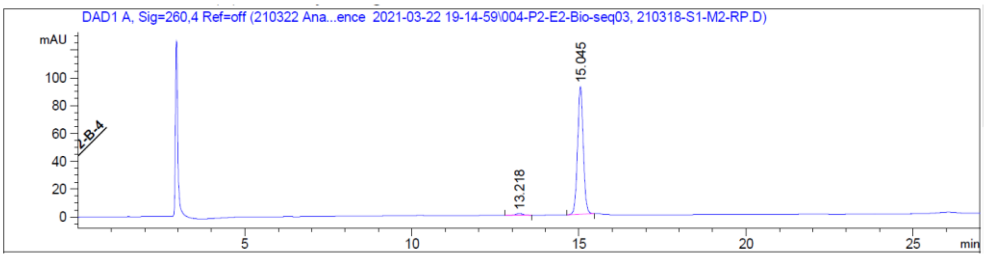

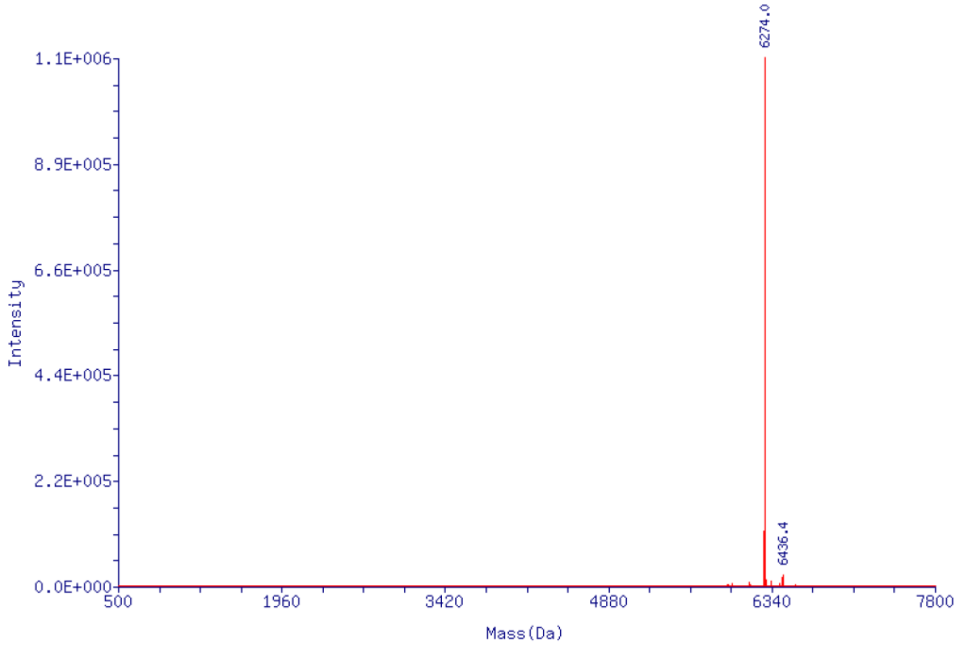

ERG-R-C-N1

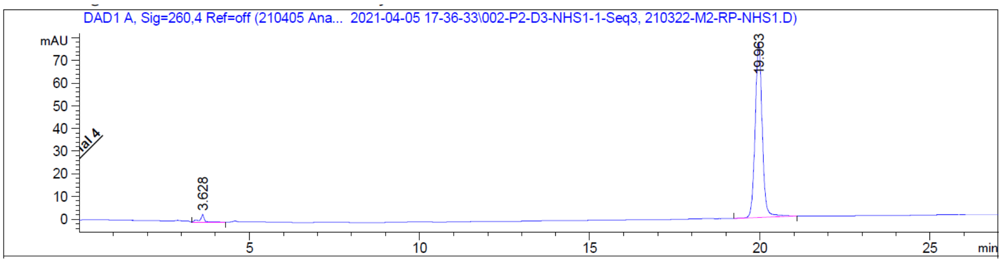

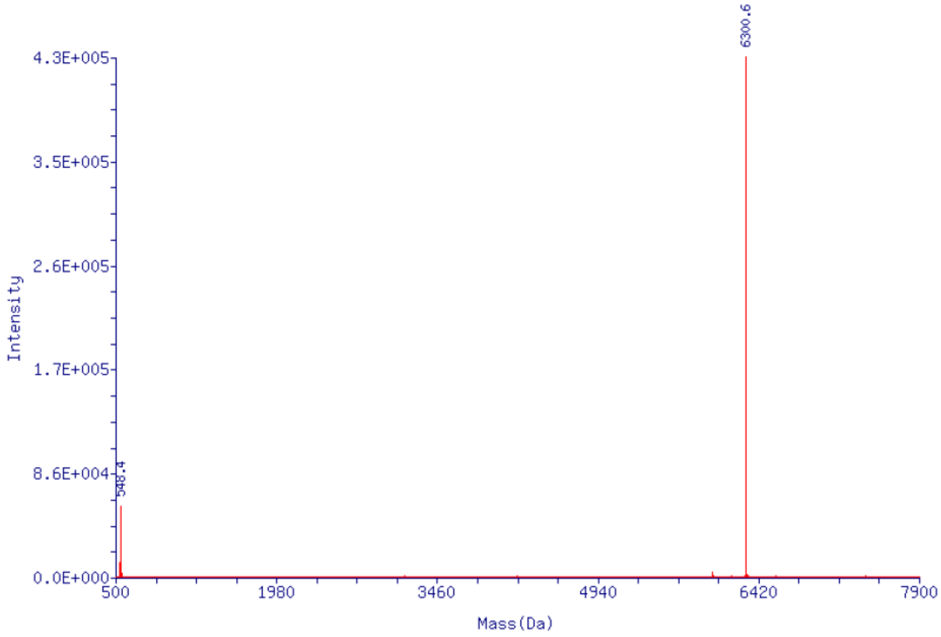

ERG-R-C-N2

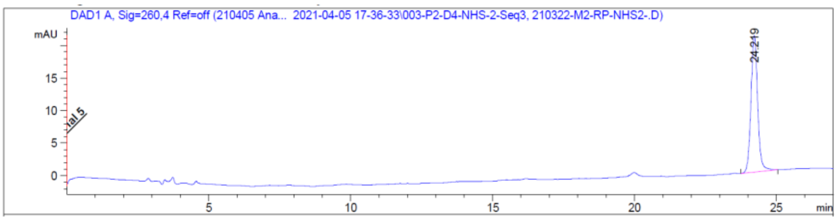

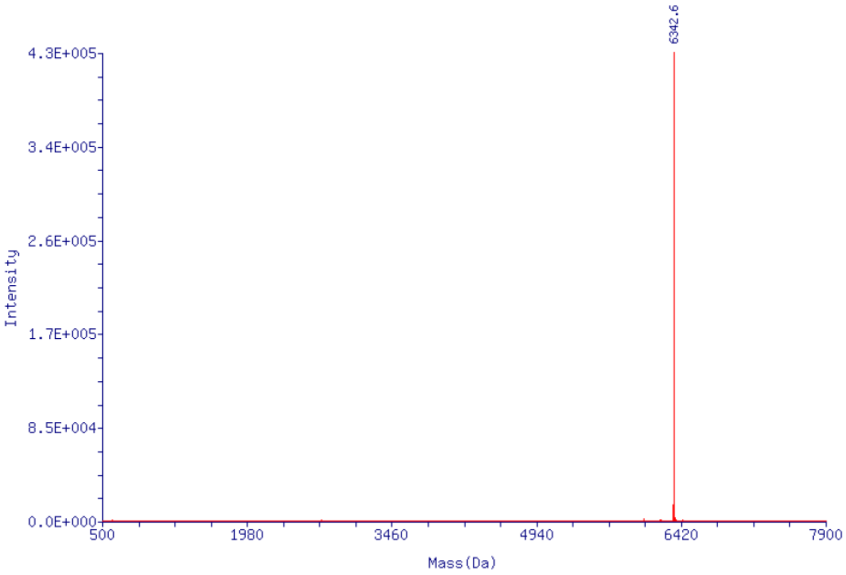

ERG-R-C-A1

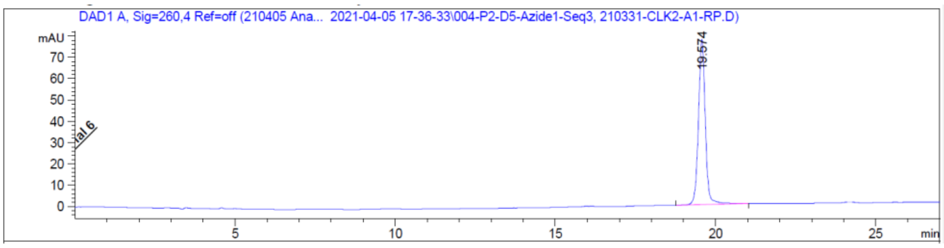
DAD1 A, Sig=260,4 Ref=off (210405 Ana... 2021-04-05 17-36-33\004-P2-D5-Azide1-Seq3, 210331-CLK2-A1-RP.D)
mAU
19.574
min

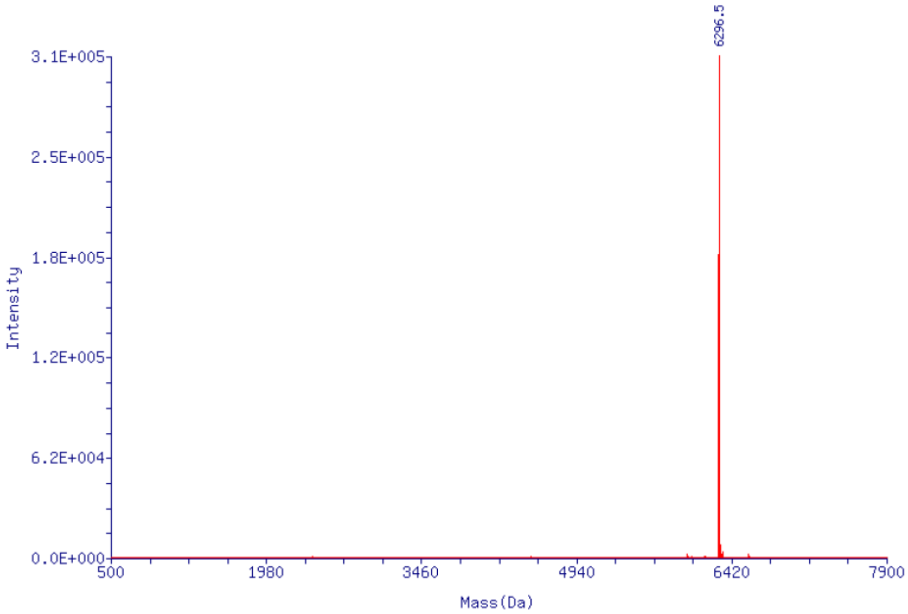
6296.5
Intensity
Mass(Da)

ERG-R-C-A2

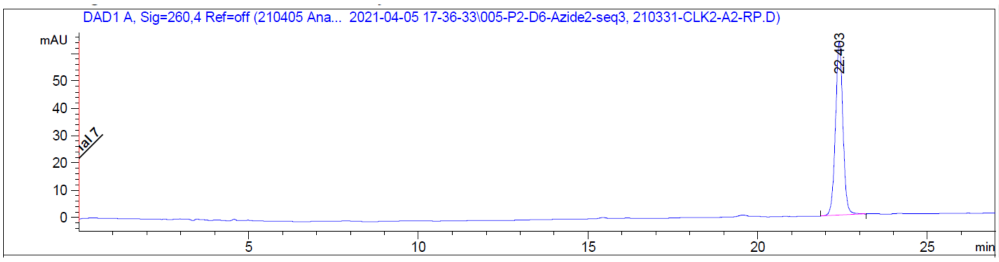
DAD1 A, Sig=260,4 Ref=off (210405 Ana... 2021-04-05 17-36-33\005-P2-D6-Azide2-seq3, 210331-CLK2-A2-RP.D)
mAU
22.403
min

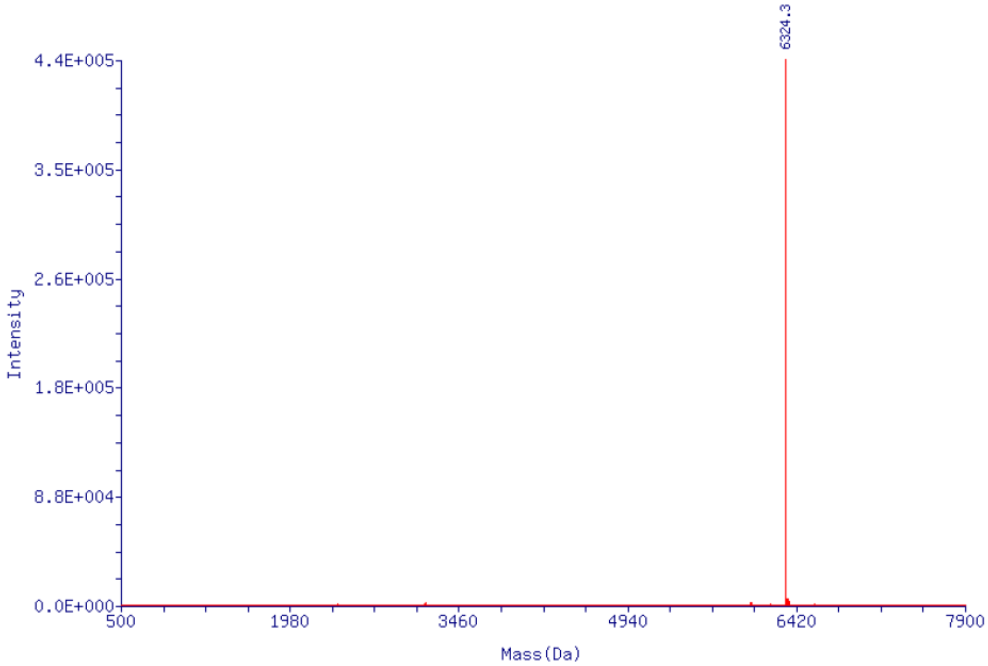

ERG-R-V1

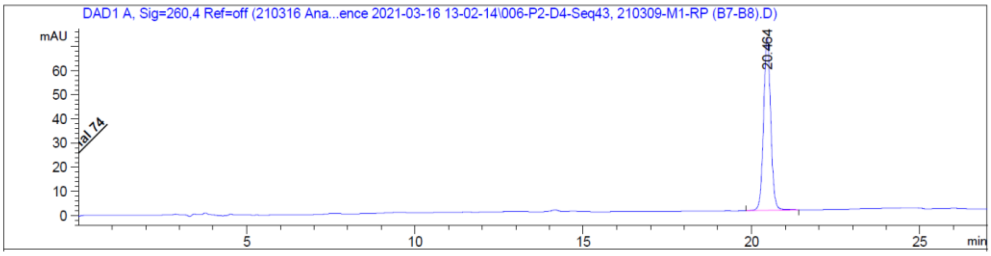

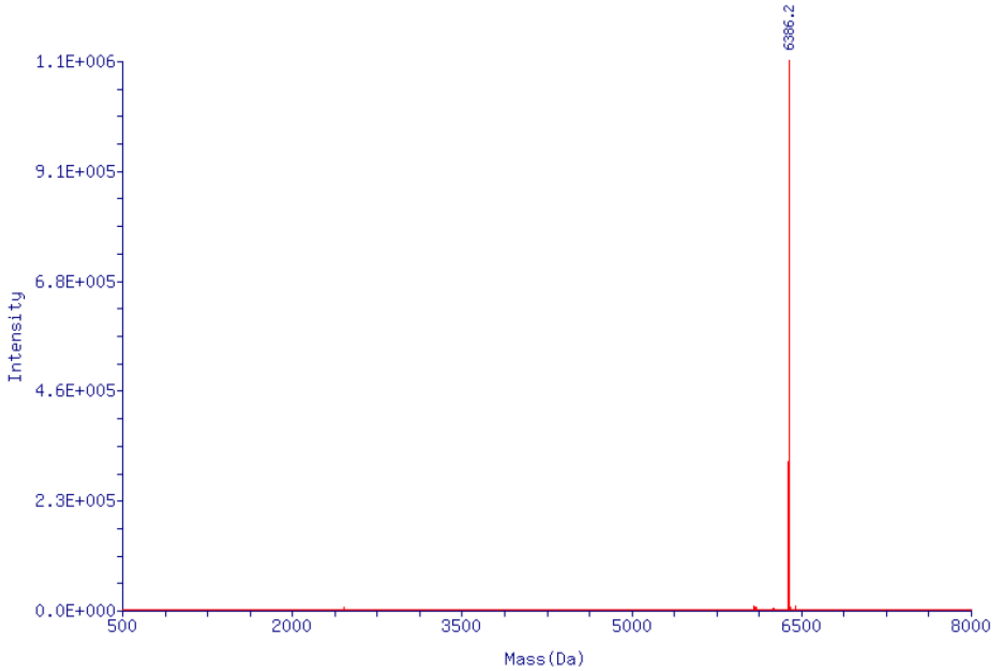

ERG-R-V2

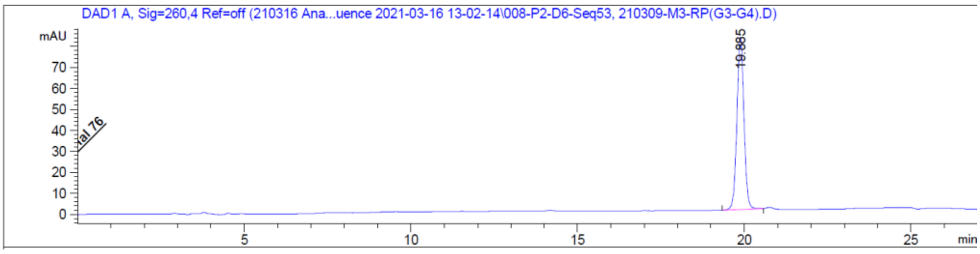
DAD1 A, Sig=260,4 Ref=off (210316 Ana...uence 2021-03-16 13-02-14\008-P2-D6-Seq53, 210309-M3-RP(G3-G4).D)
mAU
19.885
min

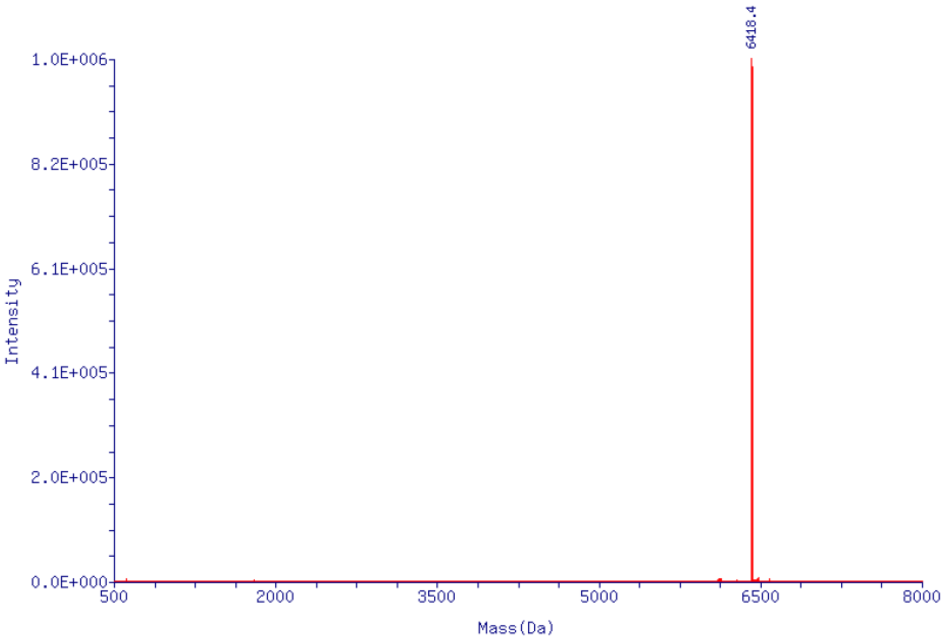
6418.4
Intensity
Mass(Da)

ERG-R-V3

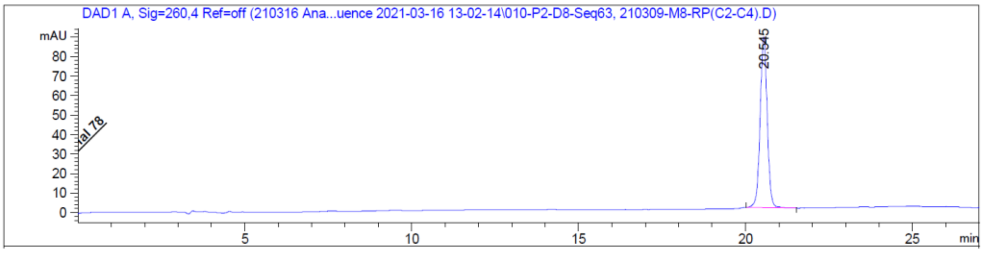
DAD1 A, Sig=260,4 Ref=off (210316 Ana...uence 2021-03-16 13-02-14\010-P2-D8-Seq63, 210309-M8-RP(C2-C4).D)
mAU
20.545
min

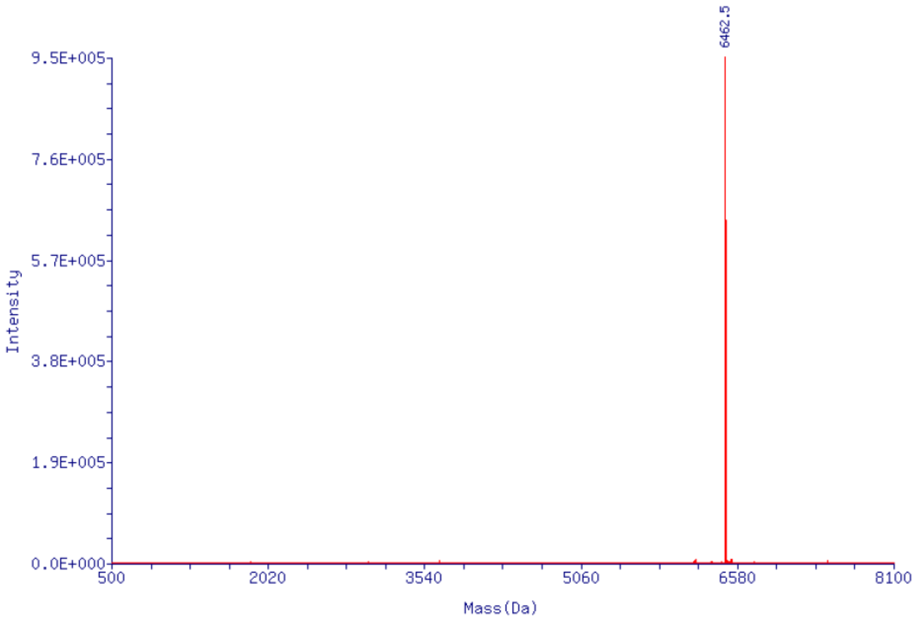
6462.5
Intensity
Mass(Da)

Control-F:

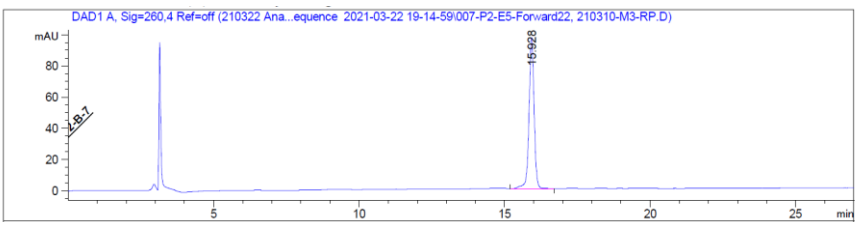

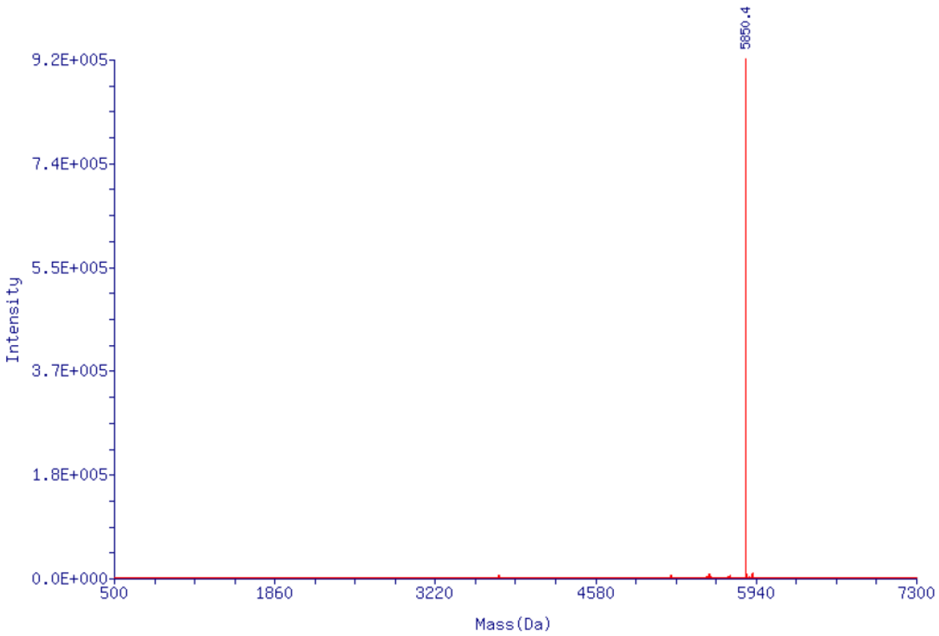

Control-R-C-N1:

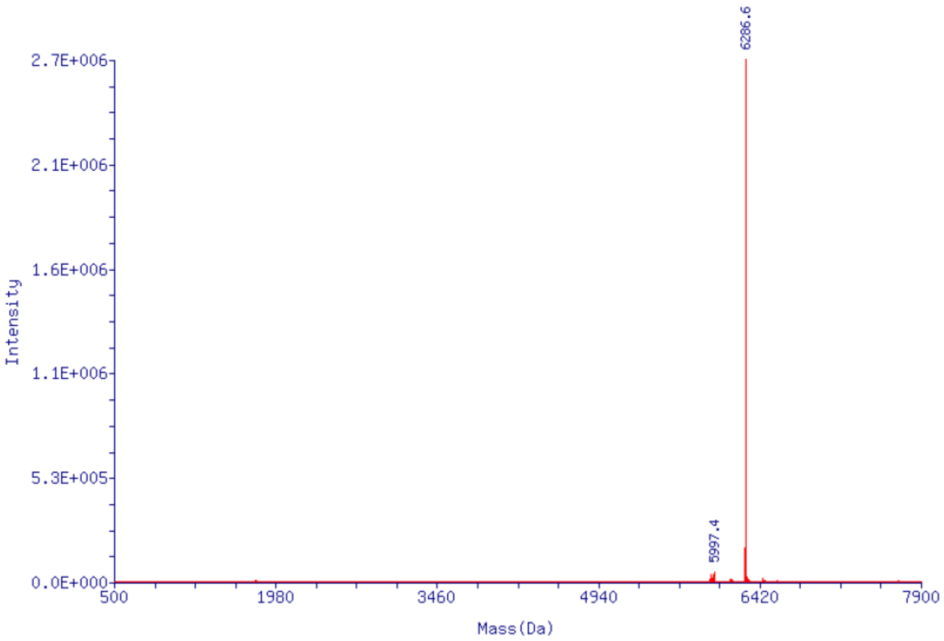

Control-R-V1:

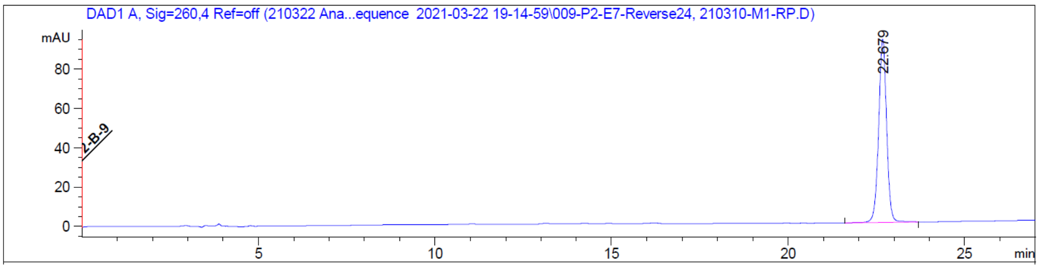

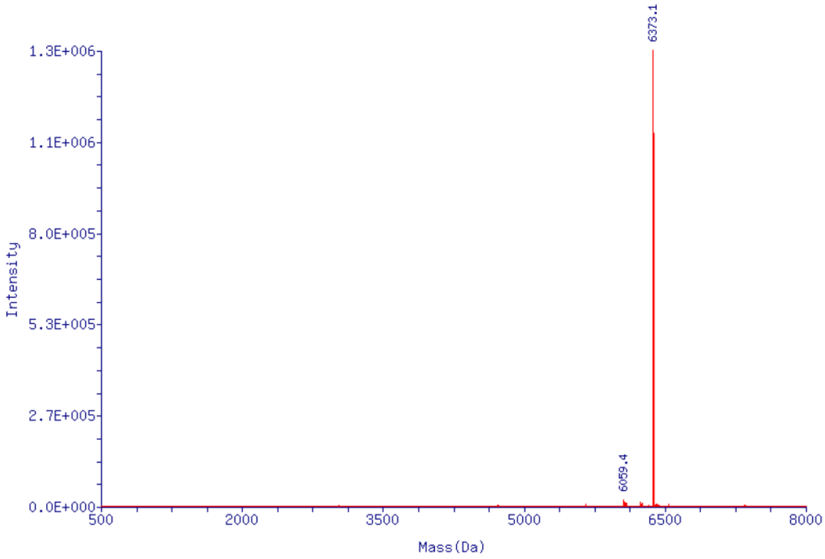

Figure S3. HPLC and MS spectrum of O'PROTACs.

References in Supporting Information

[1] a) J. Cheng, Y. Li, X. Wang, G. Dong, C. Sheng, *J. Med. Chem.* 2020, *63*, 7892; b) A. P. Crew, K. Raina, H. Dong, Y. Qian, J. Wang, D. Vigil, Y. V. Serebrenik, B. D. Hamman, A. Morgan, C. Ferraro, K. Siu, T. K. Neklesa, J. D. Winkler, K. G. Coleman, C. M. Crews, *J. Med. Chem.* 2018, *61*, 583.

[2] Z. Hong, W. Zhang, D. Ding, Z. Huang, Y. Yan, W. Cao, Y. Pan, X. Hou, S. J. Weroha, R. J. Karnes, D. Wang, Q. Wu, D. Wu, H. Huang, *Mol. Cell* 2020, *79*, 1008.
